# Which factors are associated with acquired weakness in the ICU? An overview of systematic reviews and meta-analyses

**DOI:** 10.1186/s40560-024-00744-0

**Published:** 2024-09-05

**Authors:** Rocío Fuentes-Aspe, Ruvistay Gutierrez-Arias, Felipe González-Seguel, Gabriel Nasri Marzuca-Nassr, Rodrigo Torres-Castro, Jasim Najum-Flores, Pamela Seron

**Affiliations:** 1 Departamento Ciencias de la Rehabilitación, Facultad de Medicina, Universidad de La Frontera, Claro Solar 115, Temuco, Chile; 2 Facultad de Medicina, Centro de Excelencia CIGES, Universidad de La Frontera, Temuco, Chile; 3 Departamento de Apoyo en Rehabilitación Cardiopulmonar Integral, Instituto Nacional del Tórax, Santiago, Chile; 4 Exercise and Rehabilitation Sciences Institute, Faculty of Rehabilitation Sciences, Universidad Andres Bello, Santiago, Chile; 5 INTRehab Research Group, Instituto Nacional del Tórax, Santiago, Chile; 6 School of Physical Therapy, Faculty of Medicine, Clínica Alemana Universidad del Desarrollo, Santiago, Chile; 7 Institut d’Investigacions Biomèdiques August Pi i Sunyer (IDIBAPS), Barcelona, Spain; 8 Department of Physical Therapy, Faculty of Medicine, University of Chile, Santiago, Chile; 9 Hospital Dr. Hernán Henríquez Aravena, Unidad de Paciente Crítico Adulto, Temuco, Chile; 10 Department of Physical Therapy, College of Health Sciences, University of Kentucky, Lexington, USA

**Keywords:** Intensive care unit, Muscle weakness, Risk factors, ICUAW

## Abstract

**Rationale:**

Intensive care unit-acquired weakness (ICUAW) is common in critically ill patients, characterized by muscle weakness and physical function loss. Determining risk factors for ICUAW poses challenges due to variations in assessment methods and limited generalizability of results from specific populations, the existing literature on these risk factors lacks a clear and comprehensive synthesis.

**Objective:**

This overview aimed to synthesize risk factors for ICUAW, categorizing its modifiable and nonmodifiable factors.

**Methods:**

An overview of systematic reviews was conducted. Six relevant databases were searched for systematic reviews. Two pairs of reviewers selected reviews following predefined criteria, where bias was evaluated. Results were qualitatively summarized and an overlap analysis was performed for meta-analyses.

**Results:**

Eighteen systematic reviews were included, comprising 24 risk factors for ICUAW. Meta-analyses were performed for 15 factors, while remaining reviews provided qualitative syntheses. Twelve reviews had low risk of bias, 4 reviews were unclear, and 2 reviews exhibited high risk of bias. The extent of overlap ranged from 0 to 23% for the corrected covered area index. Nonmodifiable factors, including advanced age, female gender, and multiple organ failure, were consistently associated with ICUAW. Modifiable factors, including neuromuscular blocking agents, hyperglycemia, and corticosteroids, yielded conflicting results. Aminoglycosides, renal replacement therapy, and norepinephrine were associated with ICUAW but with high heterogeneity.

**Conclusions:**

Multiple risk factors associated with ICUAW were identified, warranting consideration in prevention and treatment strategies. Some risk factors have produced conflicting results, and several remain underexplored, emphasizing the ongoing need for personalized studies encompassing all potential contributors to ICUAW development.

**Supplementary Information:**

The online version contains supplementary material available at 10.1186/s40560-024-00744-0.

## Introduction

Intensive care unit-acquired weakness (ICUAW), coined in 1984 [1], is defined as a neuromuscular condition developing during extended intensive care unit stays or as clinically detected weakness explained by critical illness as the main reason [2, 3]. The neuromuscular dysfunction of ICUAW has no clear etiology related to critical illness or the associated treatments [2]. The reported incidence of ICUAW varies widely, with studies indicating rates from 25% to as high as 100%. This variability can be attributed to factors such as the characteristics of the patient population under study and the timing of evaluations [2, 4, 5]. The diagnosis of ICUAW is based on clinical findings (Medical Research Council score, MRC), electrophysiological assessments, radiological techniques (muscle ultrasound, magnetic resonance), and, if necessary, muscle biopsies (histological and molecular analysis) [6, 7]. The signs of ICUAW include loss of muscle mass and short- and long-term physical deterioration [8]. The disorder presents with generalized, symmetrical muscle weakness, affecting the muscles of the limbs (mainly proximal) and respiratory muscles, while facial, and ocular muscles usually remain unscathed [2, 3]. The long-term effects of ICUAW can be significant and can impact a patient's quality of life, including persistent fatigue and a reduced ability to perform daily activities [6, 9].

Determining risk factors for ICUAW and neuropathies in critically ill patients poses challenges due to variations in assessment methods and limited generalizability of results from specific populations [10, 11]. The existing literature on these risk factors lacks a clear and comprehensive synthesis. The objective of this overview was to comprehensively identify and synthesize all the reported risk factors for ICUAW or similar conditions in critically ill patients.

## Methods

An overview of systematic reviews was conducted. The study protocol was registered in PROSPERO (CRD42020207863). Guidelines set forth by the JBI Collaboration [12, 13] and the PRIOR statement [14] were followed.

### Search strategy

A systematic, sensitive, and reproducible search strategy was conducted up to August 2023. The following electronic databases were searched: MEDLINE, EMBASE, CINAHL, Cochrane Library, Google Scholar, and Epistemonikos (Supplementary Table 1).

### Selection criteria and study selection

Systematic reviews with or without meta-analyses were included. Only reviews with critical methodological components, such as comprehensive search strategies and risk of bias assessment [14], were integrated into the analysis. This meticulous approach aimed to minimize selection and interpretation biases, as comprehensive searches spanned multiple databases and were unrestricted by language or publication date, enhancing the inclusivity of the study selection.

The target population consisted of adult patients hospitalized in intensive care units (ICU). Risk factors, defined as any condition or attribute that increases the likelihood of ICUAW.

As for the event of interest (outcome), systematic reviews that addressed ICUAW or similar diagnoses, such as critical illness polyneuromyopathy (CIPNM), critical illness polyneuropathy (CIP), and critical illness myopathy (CIM), were considered. Systematic reviews also eligible if they addressed any outcome related to muscular weakness. This inclusive criterion ensure that our review captured the complete spectrum of factors cited in previous systematic reviews, including biological, pre-existing and illness-associated factors.

For this overview, studies related to social or economic risk factors, or external factors such as the infrastructure of the facility, access, and availability of resources, were not considered and were excluded. This exclusion criterion was applied to maintain a focused analysis on the clinical and physiological aspects directly associated with the patient's condition and treatment within the ICU setting, thereby delineating the scope of our review to factors that are inherently linked to the patient's immediate medical care and biological responses.

The screening and review selection was independently conducted by two pairs of collaborators (RGA, RTC, GMN, FGS) using COVIDENCE® [15]. Discrepancies were resolved by a third reviewer (RFA) and a senior researcher (PS). The selection process and reasons for exclusion are presented in accordance with the PRISMA 2020 flow diagram [16].

### Data extraction, and data analysis

Data extraction carried out by the lead author (RFA) was verified by two coauthors (RGA, GMN) using a standardized data collection form. The data extracted from the selected systematic review included: year of publication, author(s), title, type of study (systematic review with or without meta-analyses), characteristics of the study population, study designs, number of primary studies included, the range of participants covered in the reviews, and the diagnostic tool used.

Both quantitative and descriptive information concerning risk factors from each systematic review was meticulously extracted. Risk factors are categorized as modifiable and nonmodifiable to underscore their potential for clinical intervention. This classification approach was derived from definitions found in the literature, particularly a narrative review led by experts in ICUAW [3]. The findings were clearly delineated, presenting each risk factor alongside its respective association outcome with ICUAW. Meta-analysis results are described only when they were conducted in the included systematic reviews. These results are detailed in the text for each meta-analysis, specifying whether or not there is an association of each risk factor with ICUAW. Additionally, the results of the heterogeneity analysis presented in the original articles are detailed. The numerical data from these analyses are also presented in the Summary Tables. For systematic reviews without meta-analyses, outcomes were described exactly as reported in the original studies, ensuring no interpretations, selections, or omissions were made. All data are comprehensively displayed in the text, tables, and figures.

A systematic review of systematic reviews should assess and report the degree of overlap of primary studies in the conducted meta-analyses [17].

In this overview, such an analysis was performed for systematic reviews with meta-analyses reporting the same risk factor. The "Corrected Coverage Area Index" (CCA) was calculated for these overlap analyses, and a heatmap was created to visualize the overlap results using the "ccaR package" [18]. The results of the overlaps for each risk factor are detailed in the text and categorized as Slight (CCA: 0–5), Moderate (CCA: 6–10), High (CCA: 11–15), or Very High (> 15), following the CCA interpretation guidelines by Pieper et al. [17]. The level of overlap is reported exclusively in the results section; it is described for informational purposes without specific strategies to address it. Further details on data extraction and analysis are provided in the supplementary material.

### Risk of bias assessment

The risk of bias was assessed using ROBIS tool [19]. Two reviewers (GMN, RGA) independently assessed bias risk, while disagreements were resolved by the senior (PS) and principal researchers (RFA). Risk of bias didn´t influence study eligibility or exclusion in this overview. A graphical representation of the bias risks was created using the templates from the "resources for ROBIS tool" at the University of Bristol [20].

## Results

**Fig. 1:**
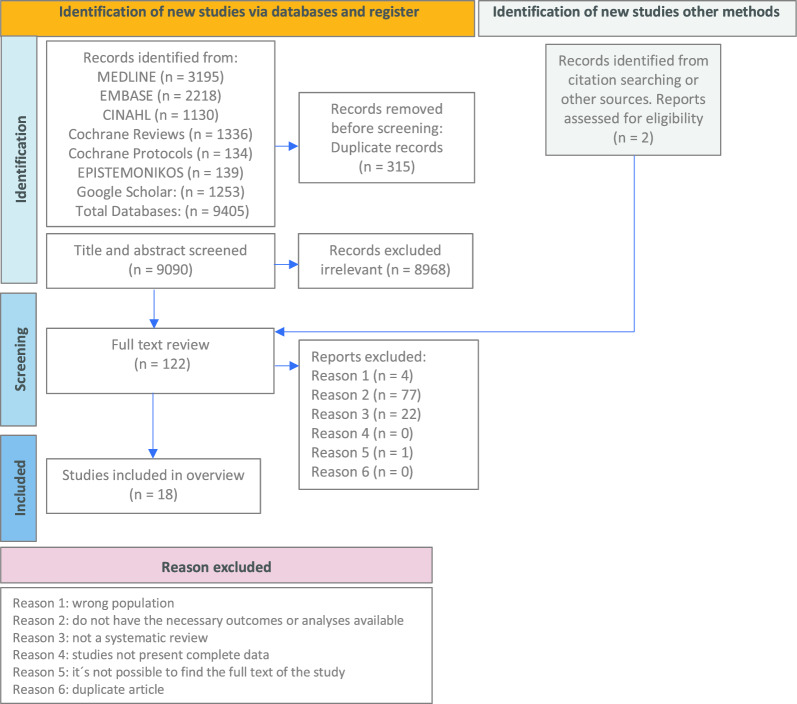
PRISMA flow diagram of study selection process

Out of 9090 titles found, screening yielded 122 reviews, of which 18 were included after full text review. (Fig. 1, PRISMA flow diagram). Of them, ten included meta-analyses [21–30] while eight were qualitative reviews of primary studies [31–36, 38, 39] (Table 1 and Supplemental material Table 1 complementary).

**Table 1:** Characteristics of the selected systematic reviews

ID Article Year_Author	Type of review / types of studies of primary studies included in each SR	N of studies included* / total patients (range of participants)	Participant details / setting and context	Risk factors or exposition reported in the selected studies*		Outcome and diagnostic tool for outcome(s) reported	Description of the main results* reported by SR **result of no meta-analysis
				Characteristic’s patient baseline at ICU admission (nonmodifiable)	Pathology´s progression status / Pharmacological or interventions (modifiable)		
2023_Bellaver [30]	SR and M-A / RCTs, observational cohort studies	30 / 3839	ICUAW patients		Use of NMBAs	Occurrence of ICUAW	NMBA use may relate to ICUAW, but evidence remains insufficient due to observational studies' heterogeneity
2022_Yang_Zi [29]	Prospective cohort research	12 / 1950 (26–474)	ICU adult patients (age ≥ 18 years old)	Sex, age, Infectious disease, SOFA score, sepsis, APACHE II, MV days, LOS	Use of aminoglycosides, RPT, use of corticosteroids, use of NMBAs, hyperglycemia	Occurrence of ICUAW	The results showed that the significant risk factors for ICUAW included female, MV days, age, ICU LOS, infectious disease, RRT, use of aminoglycosides drugs, sepsis related SOFA score, hyperglycemia. The evidence for the predictive value of glucocorticoids, MNBAS and sepsis is insufficient and needs to be validated by more high-quality studies in the future
2020_Shao [26]	SR and M-A / RCTs	3 / 691	Adult > 18 years with ARDS, study groups that received NMBAs and CG (placebo without NMBAs)	APACHEII	NMBAs	Clinical: MRC score. Occurrence of ICUAW	The use of NMBAs did not significantly increase the risk of ICUAW compared to non-NMBA treatment. Regarding severity, two studies reported significant differences in APACHE II scores between the groups, while another two studies involving 1345 patients found no statistically significant differences in MRC scores
2020_Medrinal [28]	SR and M-A / observational cohort studies	11 / 1290 (13–227)	Patients with and without weakness		Prolonged VM, prolonged ICU stay	Limb and/or respiratory muscle weakness, cut-off value based on the literature (MIP and MRC scores)	Muscle weakness was often associated with a longer duration of MV and a longer ICU LOS. Muscle weakness was strongly associated with an increase in MV weaning failure rate
2020_Tarazan [25]	SR and M-A / parallel-group RCT	4 / 885 (36–454)	Adults with ARDS of any severity		NMBA (comparing infusion of NMBA vs. non-NMBA)	ICUAW incidence	The use of NMBA infusion may increase the risk of ICU-AW. The certainty of evidence was moderate. Anticipated absolute effect (risk with no infusion, but intermittent needed NMBA)
2020_Wei [24]	SR and M-A / RCTs	4 / 1437	ARDS patients		NMBA (comparing infusion of NMBA vs. non-NMBA)	ICUAW incidence	Use of NMBA infusion was an independent risk factor might increase the risk of ICUAW which subsequently increased the duration of MV. Recent data have shown that NMBA may not be associated with ICUAW when used for less than 48 h. A continuous NMBA infusion needs to be used with caution, because it may increase the risk of ICUAW
2020_Yang3 [21]	SR and M-A / RCTs and PCS	10 / 1363 (33–412)	ICUAW patients		Role of aminoglycoside therapy	ICUAW incidence, MRC weakness scale, electrophysiological studies, and the histopathology of muscle or nerve tissue	The use of aminoglycosides was significantly associated with ICUAW. The overall incidence of ICUAW was 45% in the aminoglycoside group versus 35% in the control group
2018_Yang2 [23]	SR and M-A / PCS	14 / 2571 (39–600)	ICUAW patients with MV in 9 studies, ICU LOS in 3, SIRS or sepsis in 3 and MOF in 2	Age, APACHE II, SIRS, SOFA, neurologic failure (GCS < 10)	NMBAs, aminoglycosides, corticosteroids, norepinephrine, RRT, parenteral nutrition	Occurrence of ICUAW, MRC scale, electrophysiological studies or histopathology of muscle or nerve tissue	APACHE II, NMBAs, use of aminoglycosides were significantly associated with ICUAW (pooled data using M-A) **A number of early risk factors found to be significantly correlated with ICUAW: female sex, APACHE II score (> 12), SOFA score (> 7), higher lactate level, hyperglycemia, electrolyte disturbance, SIRS, sepsis and MOF
2018_Yang1 [22]	SR and M-A / RCTs and PCS	18 / 2387 (20–412)	Adult ICU patients	Sepsis, MV	Corticosteroids	ICUAW incidence, MRC scale or diagnostic tests, electrophysiological studies, histopathology of muscle or nerve tissue	The use of corticosteroids was significantly associated with increased odds of developing ICU-AW. The overall incidence of ICUAW was 43% in the corticosteroid group versus 34% in the control group. Thus, exposure or administration to corticosteroids should be limited or shortened in clinical practice to reduce the risk of ICUAW
2018_Sanchéz-Solana [32]	SR / retrospective observational studies, PCS or clinical trials	3 / (12–133)	CIPNM who are in the ICU	LOS	Corticosteroids	Onset of a polyneuromyopathy	**A statistically significant relationship was observed between ICUAW and failure in ventilator disconnection, increase in ICU stay and the time that the patients required MV. The use of corticosteroids, was not shown to be related to neuromuscular alteration
2018_Lambell [35]	SR /no restrictions	6 / (15–119)	Critically ill adult participants aged > 18 years admitted to an ICU		Nutrition: energy and/or protein delivery	Change in skeletal muscle mass, measured via dual-energy x-ray absorptiometry, computed tomography, ultrasound and/or TBP via prompt γ in vivo neutron activation analysis	** A variety of methods were used to assess skeletal muscle mass or TBP. Participants in included studies experienced differing levels of muscle loss (0%–22.5%) during the first 2 weeks of ICU admission. No association between energy and protein delivery and changes in skeletal muscle mass were observed. Muscle wasting is not consistent across muscle groups during the acute phase of critical illness and the difficulty with comparing findings between heterogeneous studies
2017_Annoni [39]	SR and M-A / experimental and observational studies	8–26 / 3765	Adult critically ill patients	Age, gender, severity of illness, diabetes, SOFA, score sepsis	Use of corticosteroids, vasopressor, NMBA, aminoglycosides	ICU-AW outcomes, evaluated using clinical MRC score, electrophysiological tests or a combination of both	The M-A of 8 studies (1488 patients) showed that older age, female gender, higher APACHE-II score, higher SOFA score, sepsis on admission and any use of corticosteroids during ICU stay were significantly associated with ICU-AW at awakening. History of diabetes was not associated with ICU-AW in any of 7 studies. 8 in 10 studies reported no association between aminoglycosides and ICU-AW
2017_McKittrick [34]	Integrative review / studies, case reports	7 / 2755	Patients admitted to an ICU with a severe burn injury	Severe burns		Development of critical care neuropathy, measured by EMG and NCS investigations	An analysis of 7 prospective cohort studies, with a total of 2755 burned subjects, 128 presented critical polyneuropathy, representing an incidence of 4.4%. Patients who sustain a severe burn injury are likely to have a greater length of stay in ICU thereby increasing their risk for critical care polyneuropathy
2016_Price [27]	SR and M-A / RCTs and prospective observational cohort studies	19 / 2254	Neuromuscular dysfunction acquired in critical illness, ICUAW, CIP, and CIM		NMBA, depth of sedation	Incidence ICUAW, MRC score, electrophysiologic outcomes, and use of muscle biopsy	The 19 studies included 2,254 people, showed an unadjusted event rate of neuromuscular dysfunction acquired in critical illness of 51% in patients exposed to NMBAs and 39% in the unexposed control group; this difference was statistically significant. This M-A suggests a modest association between NMBA and neuromuscular dysfunction acquired in critical illness
2012_Ydemann [31]	SR / not informed	5 / not described	CIM, CIP intensive care, ICU	LOS in ICU, length of MV	Intensive insulin therapy, minimal sedation	CIM and CIP	**CIM/CIP is the most commonly occurring ICU, ICU-acquired neuromuscular dysfunction, and it is associated with a significant increase in LOS, delayed weaning from MV, prolonged rehabilitation and, consequently more expenses
2010_Prentice [33]	SR / observational studies	19 / 116	Intensive critical care setting population	MV, sepsis and MOF	Immobility	Muscle strength (peripheral neuromuscular dysfunction, respiratory muscles)	**There appears to be a lower incidence of respiratory muscle involvement in the presence of critical illness related peripheral neuromuscular disorders. Increases in the duration of MV and ICU LOS were noted in patients with respiratory involvement compared with those without
2006_Hohl [36]	SR /not informed	6 / (50–1548)	All-encompassing term CIPNM	APACHE III, SIRS, MV	Glucose levels, use of aminoglycosides, corticosteroids, muscle relaxants	Incidence CIPNM	**Patients with elevated blood glucose levels during their ICU admission showed a higher incidence of CIPNM, correlating significantly. A high percentage (60%) of patients had CIPNM. APACHE III score and the presence of SIRS were significant predictors for the development of CIPNM, the overall incidence of CIPNM in a sample of 98 patients was 33%. No significant differences were found regarding particular drugs and the onset of CIPNM
1998_De Jonghe [38]	SR /PCS	8 / (20–242)	Critically ill adult patients	Severe asthma, MV, SOFA	Corticosteroids, NMBA	Frequency of critical illness neuromuscular abnormalities (CINMA)	** MV’s patients for more than 5 days, electrophysiologic abnormalities were reported in 76% of cases. In studies with patients with asthma and/or administration of corticosteroids and/or NMBA, 20%-50% clinical weakness or muscle denervation was observed. In 2 cohort studies of patients with SOFA, abnormalities were found whit-in 70 and 82%, the most frequent finding was axonal neuropathy. In the two other studies primary muscle disease was found in 78% of patients and was frequently associated with signs of denervation due to axonopathy

^*^Relevant for this overview and categorized, **Result of no meta-analysis, report data of primary studies or qualitative description, *ID* identificatory selected study, *N* number, *SR* systematic review, *M-A* meta-analysis, *RCTs* randomized controlled trials, vs versus, *ICUAW* intensive care unit-acquired weakness, *CIPNM* patients critical illness polyneuromyopathy, *CIP* critical illness polyneuropathy, *CIM* critical illness myopathy, *MV* mechanical ventilation, *BMI* Body mass index, *6MWT* Six minute walk test, *ICU* Intensive Care Unit, *ARDS* acute respiratory distress syndrome, *NMBA* neuromuscular blocking agents, MRC medical research council weakness scale, *APACHE II* acute physiology and chronic health disease classification system II, *APACHE III* acute physiology and chronic health disease classification system III, *GCS* Glasgow coma scale, SOFA sepsis related organ failure assessment, SIRS systemic inflammatory response syndrome, *LOS* length of stay, *RRT* renal replacement therapy, *TBP* total body protein, *EMG* electromyography, *NCS* nerve conduction studies, *MOF* multiple organ failure, *CG* control group, *PCS* prospective cohort studies

### Risk of bias analysis

**Fig. 2:**
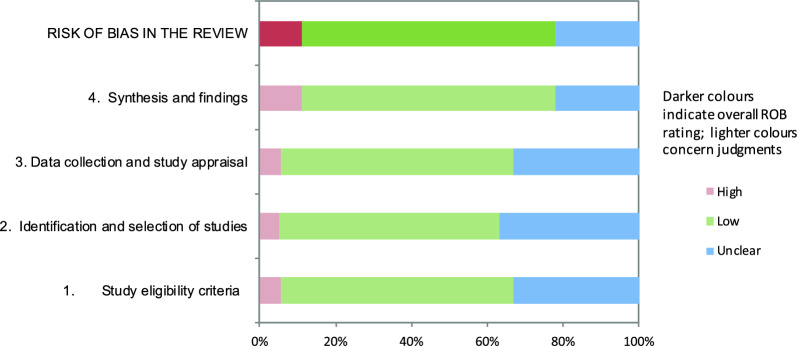
Graphical display risk of bias with ROBIS assessments

The risk of bias analysis revealed that 12 systematic reviews had a "low risk of bias" [21–30, 35, 39], 4 reviews had an "unclear risk of bias" [32, 33, 36, 38], and 2 reviews had a “high risk of bias” [31, 34]. The reviews were classified as “unclear risk of bias” or “high risk of bias” due to limitations in methodology because the language was restricted (Domain 1), the searches were limited across various databases (Domain 2), the risk of bias assessments in the primary studies was inadequate (Domain 3), or the reviews failed to address the biases in primary studies (Domain 4). Most reviews with a higher or uncertain risk of bias were published before 2012. Table 2 shows the ROBIS assessments, and Fig. 2 shows the overall risk of bias ratings.

**Table 2:** ROBIS assessments

Review	Phase 2				Phase 3
	1. Study eligibility criteria	2. Identification and selection of studies	3. Data collection and study appraisal	4. Synthesis and findings	Risk of bias in the review
2023_Bellaver	Low risk	Low risk	Low risk	Low risk	Low risk
2022_Yang Zi	Low risk	Low risk	Low risk	Low risk	Low risk
2020_Shao	Low risk	Low risk	Low risk	Low risk	Low risk
2020_Medrinal	Low risk	Unclear risk	Low risk	Low risk	Low risk
2020_Tarazan	Unclear risk	Low risk	Unclear risk	Low risk	Low risk
2020_Wei	Unclear risk	Unclear risk	Low risk	Low risk	Low risk
2020_Yang3	Low risk	Low risk	Low risk	Low risk	Low risk
2018_Yang2	Unclear risk	Unclear risk	Low risk	Low risk	Low risk
2018_Yang1	Unclear risk	Low risk	Low risk	Low risk	Low risk
2018_Sanchéz-Solana	Unclear risk	Low risk	Unclear risk	Unclear risk	Unclear risk
2018_Lambell	Low risk	Low risk	Low risk	Low risk	Low risk
2017_Annoni	Low risk	Low risk	Low risk	Low risk	Low risk
2017_McKittrick	Low risk	Unclear risk	Unclear risk	High risk	High risk
2016_Price	Low risk	Low risk	Low risk	Low risk	Low risk
2012_Ydemann	Unclear risk	Unclear risk	High risk	High risk	High risk
2010_Prentice	Low risk	Low risk	Unclear risk	Unclear risk	Unclear risk
2006_Hohl	High risk	Unclear risk	Unclear risk	Unclear risk	Unclear risk
1998_De Jonghe	Low risk	Unclear risk	Unclear risk	Unclear risk	Unclear risk

### Risk factors for ICUAW

**Fig. 3:**
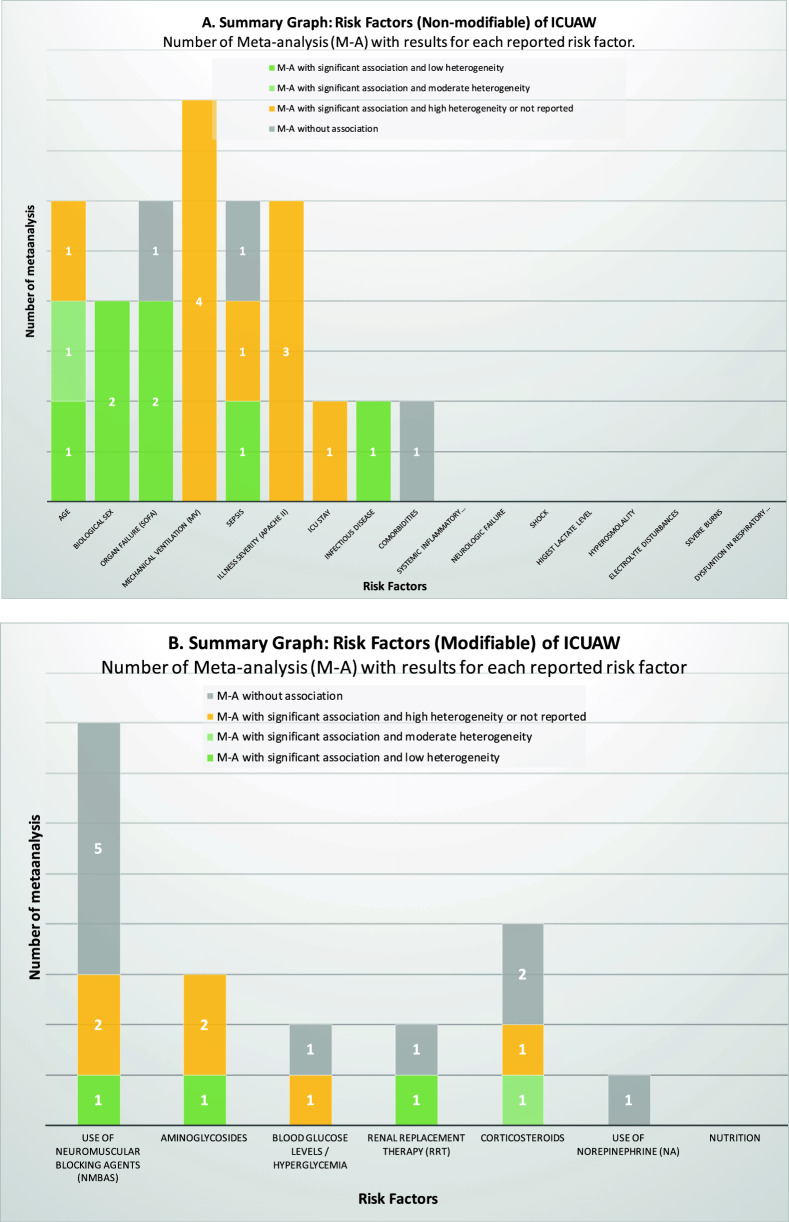
Summary of risk factors. **A** Risk factors (nonmodifiable) of ICUAW. **B** Risk factors (modifiable) of ICUAW

**Fig. 4:**
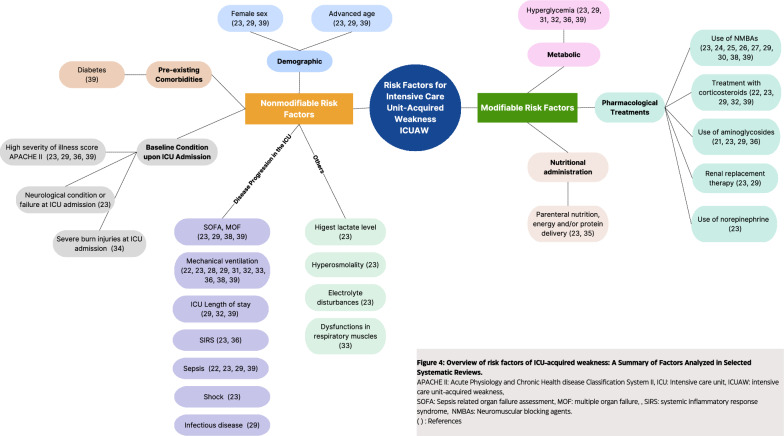
Overview of risk factors of ICUAW

Twenty-four risk factors for ICUAW were identified, predominantly nonmodifiable, including sex, age, and severity of pathology upon ICU admission, among others. Modifiable factors that were identified include hyperglycemia, use of neuromuscular blocking agents (NMBAs), corticosteroid treatment, aminoglycoside use, renal replacement therapy (RRT), and norepinephrine (NA) use. All factors identified across the systematic reviews are detailed and schematized in Figs. 3 and 4. Tables 3 and 4 describe the magnitude of the association of each risk factor with ICUAW.

**Table 3:** Summary of nonmodifiable factors

Baseline patient characteristic (nonmodifiable) Pre-existing, comorbidities and baseline conditions upon ICU admission	Detail or specification / number of studies	ID_study year_author	Results / findings with M-A; Heterogeneity	Results / findings without M-A	Synthesized finding / conclusion for the reported outcome
Biological sex [23, 29, 39]	Female / M-A of 9 studies	2022_Yang_Zi	OR 1.34, 95% CI (1.06–1.71), *p* = 0.02; *I*^2^ = 16%, *p* = 0.30		Female sex were significantly associated with ICUAW
	Female / M-A of 4 studies, 19 individual studies	2017_Annoni	OR 1.6 95% CI (1.22–2.14); I^2^ = 0% *p* = 0.46		M-A of 4 studies shows a significant association. Low heterogeneity. Female gender was associated with ICUAW in 5 of 19 individual studies
	Female / 1 study	2018_Yang_2	Not M-A	OR 4.66 95% CI (1.19–18.30) *p* = 0.02	Associated with increased odds of developing ICUAW on multivariate analysis in each single studies
Age [23, 29, 39]	Age / M-A of 8 studies	2022_Yang_Zi	MD 6.33 95% CI (5.05–7.61); *p* < 0.00001; *I*^2^ = 50%, *p* = 0.06		MD age was higher in individuals with ICU-AW, and it was statistically significant
	Age, M-A of 5 studies	2018_Yang_2	OR 1.01; 95% CI (0.99–1.03); *I*^2^ = 82.4%		There was no significant association found between age and ICUAW based on the overall effect size. High heterogeneity
	M-A of 5 studies, 19 individual studies	2017_Annoni	MD 3.46 95% CI (0.94–5.98); *I*^2^ = 18% *p* = 0.30		M-A shows a significant association (low heterogeneity). 4 of 19 studies reported a positive association between older age and ICUAW
Comorbidities [39]	Diabetes / M-A of 2 studies, 7 individual studies	2017_Annoni	OR 1.27; 95% CI (0.75–2.15); *I*^2^ = 22%, *p* = 0.26		History of diabetes was not associated with ICUAW in any of seven studies or in M-A of 2 studies. Low heterogeneity
Severity of illness at ICU admission: APACHE [23, 29, 36, 39]	APACHE II / M-A of 9 studies	2022_Yang_Zi	MD 4.78 CI 95% (1.96–7,60), *I*^2^ = 93%, *p* = 0.0009		M-A shows association with very high heterogeneity
	APACHE II / M-A of 5 studies	2018_Yang_2	OR 1.05 CI 95% (1.01–1.10); *I*^2^ = 79%		The overall effect size demonstrated a statistically significant association of APACHE II with ICUAW, with high heterogeneity
	APACHE II / M-A of 3 studies, 20 individual studies	2017_Annoni	MD 3.52 CI 95% (1.47–5.57); *I*^2^ = 64%, *p* = 0.06		M-A shows a significant association, with high heterogeneity. Severity of illness were positively associated with ICUAW in 12 of 20 studies
	APACHEII > 15 / 1 study	2018_Yang_2	Not M-A	RR 11.6 CI 95% ( 4.9–27.2)	The result of an individual study indicates that an APACHEII > 15 is associated with ICUAW
	APACHE III and SIRS / 1 study	2006_Hohl	Not M-A	Overall incidence of CIPNM was 33%	APACHE III score and the presence of SIRS were predictors for the development of CIPNM. The overall incidence of CIPNM in this sample of 98 patients was 33%
Systemic inflammatory response syndrome: SIRS [23, 36]	SIRS / M-A of 3 studies	2022_Yang_Zi	OR 2.24 CI 95% (0.57–9.56); *I*^2^ = 81%		Not found consistent evidence that sepsis have any effect on ICUAW risk
	SIRS	2018_Yang_2	Not M-A	OR 3.75 CI (1.59–8.86) *p* = 0.003	Result of an independent study, were significantly associated with ICUAW
	SIRS 1 week (d) > 3	2018_Yang_2	Not M-A	RR 3.74 CI (1.37–10.2) *p* < 0.05	Were regarded as significant risk factors for ICUAW development based on multivariate analysis of one single study
	SIRS and APACHE III	2006_Hohl	Not M-A		A study categorized patients based on the presence of SIRS to determine risk groups for developing CIPNM. The risk categories for assessing patients level of risk are as follows: high risk (72%): initial APACHE III score ≥ 85 and SIRS, medium risk (28%): APACHE III score 71–84, low risk (8%): APACHE III score ≤ 70 and absence of SIRS
Sepsis [22, 23, 29, 39]	Sepsis / M-A of 3 studies	2022_Yang_Zi	OR 1.27, IC 95% (0.41–3.96) *p* = 0.67; *I*^2^ = 77%, *p* = 0.04		Reviews found significant heterogeneity in the results. Sensitivity analyses had minimal impact on overall estimates and did not eliminate heterogeneity
	Sepsis and corticosteroids / M-A of 4 studies	2018_Yang_1	OR 1.96 CI 95% (0.61–6.30) *p* = 0.6260; *I*^2^ = 80.8% *p* = 0.001		Four trials with sepsis participants reported an association between the use of corticosteroids and ICUAW, and demonstrated an incidence of 34% in the corticosteroid group and 30% in the control group. The pooled effect revealed no significant association, with high heterogeneity
	Sepsis (on admission) / M-A of 3 studies	2017_Annoni	OR 1.48; CI 95% (1.09–2.00); *I*^2^ = 0%, *p* = 0.62		A M-A shows significative association, with low heterogeneity. Sepsis on admission were positively associated with ICUAW in two of 11 studies
	Sepsis / 1 study	2018_Yang_2	Not M-A	OR, 2.20 CI 95% (1.30–3.71) *p* < 0.05	Result of an independent study, were significantly associated with ICUAW
	Days with sepsis / 1 study	2018_Yang_2	Not M-A	HR, 1.48 CI 95% (1.22–1.81) *p* < 0.05	Result of an independent study, were significantly associated with ICUAW
Organ Failure: Sequential organ failure assessment: SOFA, Multiple organ failure: MOF [23, 29, 38, 39]	SOFA / M-A of 2 studies	2022_Yang_Zi	OR 1.07, 95% CI: 0.24–1.90; *p* = 0.01; *I*^2^ = 0% *p* = 0.44		The combined effect was statistically significant with ICUAW
	SOFA / M-A of 2 studies	2018_Yang_2	OR 0.99; CI 95% (0.92–1.08); *I*^2^ = 6.6% *p* = 0.301		M-A reveals no significant association
	SOFA / M-A of 4 studies, 13 individual studies	2017_Annoni	MD 1.96; 95% CI (1.41–2.50); *I*^2^ = 0% *p* = 0.77		M-A reveals significant association, with low heterogeneity. Also SOFA were positively associated with ICUAW in five of 13 studies
	MOF SOFA > 7 score / 1 study	2018_Yang_2	Not M-A	RR, 2.03 CI 95% (1.02–4.12), *p* < 0.05	Individual studies indicated that a SOFA > 7 were independent risk factors for ICUAW
	MOF SOFA > 45 score / 1 study	2018_Yang_2	Not M-A	RR 2.38; 95% CI (1.02–5.53) *p* < 0.05	Individual studies indicated that a total SOFA score during the first week > 45 were independent risk factors for ICUAW
	Organ failure / 4 individual studies	1998_DeJonghe	Not M-A	Not data	Two studies reported abnormalities in 70% and 82% of patients, with axonal neuropathy being the most prevalent in CIP. In the other two studies, primary muscle disease was observed in 78% of patients, often accompanied by signs of denervation related to axonopathy
Shock [23]		2018_Yang_2	Not M-A	OR 2.58; CI 95% (1.02–6.51) *p* = 0.045	Result of an independent study, were significantly associated with ICUAW
Infectious disease [29]	M-A of 4 studies	2022_Yang_Zi	OR 1.67, 95% CI (1.20–2.33) *p* = 0.002; *I*^2^ = 0% *p* = 0.002		M-A of 4 studies a reveals significant association, with low heterogeneity
Neurologic failure [23]	Neurologic failure (GSC < 10) / 1 study	2018_Yang_2	Not M-A	OR 24.02 CI 95% (3.68–156.7) *p* = 0.001	Result an individual study reveal an association between ICUAW and neurological failure (correlated with the GCS sub score of the SOFA)
Mechanical ventilation [22, 23, 28, 29, 31, 33, 36, 38, 39]	Duration of MV (days) / M-A of 5 studies	2022_Yang_Zi	OR 2.73 CI 95% 1.65 a 3.80 *p* < 0.00001; *I*^2^ = 76% *p* = 0.005		The M-A result indicates an association between MV and ICU-AW, but significant heterogeneity was observed. Sensitivity studies excluding trials with a relatively small sample size showed no significant change in the overall estimate, but heterogeneity persisted
	Duration of MV (days) / M-A of 11 studies	2020_Medrinal	Standard MD 0.69 CI 95% (0.50–0.87); *I*^2^ = 57.28%		Muscle weakness was often associated with a longer duration of MV and a longer ICU LOS
	MV and corticosteroids / M-A of 12 studies	2018_Yang_1	OR 2.00 CI 95% (1.23–3.27) *p* = 0.006; *I*^2^ = 66.0%		Twelve studies using MV and use of corticosteroids showed an event rate of 50% in the corticosteroid group and 40% in the control group. The overall effect size: Significant association, random effects model, considering heterogeneity
	Duration of MV (days) / M-A of 5 studies	2017_Annoni	MD 4.50 95% CI (2.00–7.01); *I*^2^ = 85% *p* < 0.0001		11 out of 15 studies showed a positive association with ICUAW, Duration of MV in ICUAW patients: 2–33 days v/s 1–18 days, with high heterogeneity
	Duration of MV / 1 study	2018_Yang_2	Not M-A	OR 1.10 CI 95% (1.00–1.22) *p* = 0.049	Results of a multivariate analysis of a single independent study indicate an association with increased odds of developing ICUAW
	Duration of MV and LOS / 2 individual studies	2012_Ydemann	Not M-A		In the analysis of 2 studies, CIPNM significantly increases the length of MV and the lengths of ICU and hospital stays. In patients with CIPNM and MV for more than seven to ten days, the mortality increases from 19–56.5% to 48–84%
	Duration of MV (days) and ICU LOS / 3 individual studies	2010_Prentice	Not M-A		No significant differences in the duration of MV, ICU LOS, and weaning time were found among patients with CIP based on various measures in the analysis of three independent studies
	Duration of MV (days) / 1 study	2006_Hohl	Not M-A		The results of an independent study indicate that the probability of developing CIPNM within 30 days of artificial ventilation varied from 8% in the low-risk group to 72% in the high-risk group
	Duration of MV (days) / 3 individual studies	1998_DeJonghe	Not M-A		In a report of three independent studies on a population of patients ventilated for over 5 days, 76% showed electrophysiologic abnormalities. Two of the studies demonstrated a significant increase in MV duration (5 and 9 days) and double the mortality rate in patients with critical illness neuromuscular abnormalities compared to those without
ICU Length of stay (ICU-LOS) [29, 32, 39]	M-A of 5 studies	2022_Yang_Zi	MD 3.78 CI 95% (2.06–5.51); *I*^2^ = 88%, *p* < 0.0001		Sensitivity analysis revealed a significant association between the explored factors, accompanied by notable heterogeneity
	6 individual studies	2017_Annoni	Not M-A	MD 8.60 CI 95% (4.72–12.48), *I*^2^ = 85% *p* = 0.00001	ICU LOS of ICUAW patients ranged from 6 to 41 days and from 4 to 28 days in patients without ICUAW. 17 studies reported that patients with ICUAW stayed in ICU longer than patients without ICUAW
	5 individual studies	2017_Sanchéz-Solana	Not M-A		Mean ICU LOS was generally higher for patients with CIPNM than those without, as seen in 5 primary studies. However, one study showed a slightly longer ICU stay for patients without neuromuscular changes, but the association was not statistically significant
Others: Highest lactate level [23]	1 study	2018_Yang_2	Not M-A	OR 2.18 CI 95% (1.3–3.43) *p* < 0.05	Results of a multivariate analysis of a single independent study indicate an association with increased odds of developing ICUAW
Others: Hyperosmolality [23]	1 study	2018_Yang_2	Not M-A	OR 4.8, 95% CI (1.05, 24.38) *p* = 0.046	Results of a multivariate analysis of a single independent study indicate an association with increased odds of developing ICUAW
Others: Electrolyte disturbances [23]	1 study	2018_Yang_2	Not M-A	OR 2.48; 95% CI (1.02, 6.01) *p* = 0.044	Result of an independent study, were significantly associated with ICUAW
Others: Severe Burns Injury [34]	7 individual studies	2017_Mc Kittrick	Not M-A	Incidence %: 4.4% gender: 71% males, age mean: 39,7 years	Analysis of 7 PCS with 2755 burned subjects revealed a 4.4% incidence of critical polyneuropathy. Severe burn injury increases ICU stay and risk of polyneuropathy
Others: Dysfunctions in respiratory muscles [33]	11 individual studies	2010_Prentice	Not M-A		The 11 analyzed studies showed milder respiratory muscle dysfunction compared to peripheral muscles in critically ill patients. One study found that low MIP, low MEP (< 30 cm H2O), and a low MRC sum score (< 41) independently predict delayed successful extubation for 7 or more days (8.02, 4.14, and 3.03 times higher risk, respectively)

*ID* identification, *I*^*2*^ Heterogeneity, *N/R* not reported, *SR* systematic review, *M-A* meta-analysis, *MD* mean difference, *OR* odd ratio, *RR* relative risk, *CI* confidence interval, *6MWT* six minute walk test, *ICU* Intensive care unit, *ICUAW* intensive care unit-acquired weakness, *BMI* Body Mass Index, *APACHE II* Acute Physiology and Chronic Health disease Classification System II, *APACHE III* acute physiology and chronic health disease classification system III, *CIP* critical illness polyneuropathy, *CIPNM* patients critical illness polyneuromyopathy, *SIRS* systemic inflammatory response syndrome, *PCS* prospective cohort studies, *GCS* Glasgow coma scale, *SOFA* Sepsis related Organ Failure Assessment, *MV* mechanical ventilation, *ICU-LOS* Intensive care unit length of stay, *MIP* maximal inspiratory pressure, *MEP* maximal expiratory pressure

**Table 4:** Summary of modifiable factors

Pharmacological or medical measures (modifiable)	Detail or specification / number of studies	ID_study year_author	Results/ findings with M-A; Heterogeneity	Results / findings without M-A	Synthesized finding / Conclusion for the reported outcome
Hyperglycemia [23, 29, 31, 32, 36, 39]	Hyperglycemia / M-A of 3 studies	2022_Yang_Zi	OR 1.55, CI 95% (0.47–5.12); *p* = 0.47; *I*^2^ = 80%, *p* = 0.02		The first analysis revealed significant heterogeneity in the association between hyperglycemia and the outcome
	Hyperglycemia (subgroup) / M-A of 2 studies	2022_Yang_Zi	OR 2.95 CI 95% (1.70–5.11), *p* = 0.0001; *I*^2^ = 0% *p* = 0.82		After excluding the study with the largest sample size, substantial changes in overall estimates were observed, and no heterogeneity was found between studies
	Serum glucose / M-A of 3 studies	2017_Annoni	OR 3.33 95% CI (− 6.19, 12.84); *I*^2^ = 80% *p* = 0.007		Serum glucose were not associated with ICUAW, significant statistical heterogeneity was found for serum glucose
	Hyperglycemia / 1 study	2018_Yang_2	Not M-A	OR 2.86 CI 95% (1.301–6.296) *p* = 0.009	Results of a multivariate analysis of a single independent study indicate an association with increased odds of developing ICUAW
	Administration of insulin and the measurement of glycaemia / 2 studies	2017_Sanchéz-Solana	Not M-A	CIPNM incidence: 10% vs. 45% (control) CIPNM incidence: 31% (insulin treatment) vs. 47% (control)	Two articles describe maintaining glycemic control and/or the use of insulin and its association with CIPNM, and these studies include early mobilization therapy in the analysis. Both studies showed a significant decrease in the rate of CIPNM and time on mechanical ventilation
	Intensive insulin therapy / 3 studies	2012_Ydemann	Not M-A	(1) OR of 0.49 *p* < 0.0001 (2) CIPNM incidence from 50.5% to 38.9% *p* = 0.02 with IIT (3) Reduced diagnosed CIPNM from 74.4 to 48.7% *p* < 0.0001	Report of 3 different studies: (1) Pooled data showed that IIT reduced the risk of developing CIPNM. (2) Another study demonstrated a decrease in CIPNM incidence with IIT. (3) Implementation of IIT in two ICUs also significantly reduced diagnosed CIPNM in long-stay patients
	Glucose levels	2006_Hohl	Not M-A	Strict blood glucose control (< 6.1 mmol/L) significantly reduced CIPNM incidence from 49 to 25%	Patients with HBG levels in the ICU had a higher incidence of CIPNM, affecting 60% of patients. However, a blood glucose level > 9.4 mmol/L was a positive predictor of paresis, but had low sensitivity (44%) for ruling out CIPNM
Use of Neuromuscular blocking agents (NMBAs) Deep sedation (Ramsay score of 6, RASS score of 0 to – 1) [23–27, 29, 30, 38, 39]	Use of NMBAs / M-A of 30 studies	2023_Bellaver	OR 2.77 CI 95% (1.98–3.88); *I*^2^ = 62%, *p* < 0.00001		Summarized data stratified to RCTs, observational studies and all studies. The size of the effect indicated increased odds of developing ICU-AW According to the GRADE approach, there is a low level of certainty of the evidence
	Use of NMBAs / M-A of 5 studies	2022_Yang_Zi	OR 1.43 CI 95% (0.92–2.22); *I*^2^ = 0%, *p* = 0.11		Fixed effects model and the combined effect was not statistically significant reported no significant association between NMBAs and ICUAW
	NMBAs, deep sedation / M-A of 4 studies	2020_Wei	RR 1.34 CI 95% (0.98–1.84); *I*^2^ = 0%, *p* = 0.898		The incidence of ICUAW was higher in patients who received NMBA treatment. Infusion of NMBA might increase the risk of ICUAW
	NMBAs, deep sedation / M-A of 4 studies	2020_Tarazan	RR 1.16 CI 95%; (0.98–1.37); *I*^2^ = 0%, *p* = 0.08		NMBA infusion increases ICUAW risk; however, the 95% CI includes no difference. Moderate certainty of evidence, with an anticipated absolute effect of 346 per 1000 and a risk difference of 55 per 1000
	Use of NMBAs / M-A of 3 studies	2020_Shao	RR 1.19 IC 95% (0.99–1.44); *I*^2^ = 0%, *p* = 0.07		Three studies involving 691 patients provided data on ICUAW. NMBAs did not increase the occurrence of ICU-AW compared to non-NMBA treatment
	(subgroup MRC score) M-A of 2 studies	2020_Shao	MD − 2.24 CI 95% (− 6.24–1.76) *p* = 0.27; *I*^2^ = 84%		Two studies included 1345 patients reported the MRC score. No statistically significant difference between the two groups (NMBAs experimental v/s placebo) in terms of the MRC scores
	Use of NMBA / M-A of 5 studies	2018_Yang_2	OR, 2.03 CI 95% (1.22–3.40); *I*^2^ = 72.9% *p* = 0.005		A significant association was demonstrated between NMBA use and ICUAW
	Use of NMBA / M-A of 3 studies	2017_Annoni	OR 1.61 CI 95% (0.76–3.40); *I*^2^ = 74% *p* = 0.02		Use of neuromuscular NMBA during ICU stay showed a positive association with ICUAW in 4 of 13 studies, and in M-A of 3 studies, with high heterogeneity
	Use of NMBAs / M-A of 19 studies	2016_Price	OR, 1.25 IC 95% (1.06–1.48); *I*^2^ = 16% *p* = 0.26		The pooled analysis showed a significant difference in neuromuscular dysfunction: 51% in exposed patients and 39% in controls, with low heterogeneity. The funnel plot suggests possible reporting bias due to small studies with strong associations
	Use of NMBAs (subgroup lowest RoB studies / 5 studies)	2016_Price	OR, 1.31 CI 95% (0.91–1.86); *I*^2^ = 48% *p* = 0.10		To show the pooled effect size of studies with the lowest risk of bias (1 RCT, 4 observational studies). The pooled OR was not statistically significant
	NMBA and sepsis / M-A of 2 studies	2016_Price	OR 5.36 CI 95% (1.56–18.46); *I*^2^ = 1%		The M-A of two studies (139 patients with severe sepsis or septic shock) found 83% event rate in exposed vs. 57% in unexposed group. This subgroup had a significant pooled effect size and odds ratio, with minimal heterogeneity
	NMBAs and asthma / 2 individual studies	1998_DeJonghe	Not M-A		Two studies involved patients with asthma and/or vecuronium administration. EMG measurement was not systematic, but one study showed a myopathic pattern, and the other found denervation signs in 50% of patients. Prolonged neuromuscular blockade likely contributed to weakness in 20% of patients in the latter study
Treatment with corticosteroids [22, 23, 29, 32, 39]	Treatment with corticosteroids / M-A of 8 studies	2020_Yang_Zi	OR 1.54 CI 95% (0.77–3.09); *I*^2^ = 77% *p* = 0.23		The use of corticosteroids showing significant heterogeneity. Sensitivity analysis did not substantially change overall estimates and heterogeneity persisted
	Treatment with corticosteroids / M-A of 4 studies	2018_Yang_2	OR 1.92 95% CI (0.95–3.88) *p* > 0.05; *I*^2^ = 87.2% *p* < 0.001		The effect size analysis reported no significant association between corticosteroids and ICUAW
	Treatment with corticosteroids / M-A of 18 studies	2018_Yang_1	OR 1.84 95% IC (1.26–2.67) *p* = 0.002; *I*^2^ = 67.2% *p* > 0.001		The use of corticosteroids was significantly associated with increased odds of developing ICUAW. The overall incidence of ICUAW was 43% in the corticosteroid group versus 34% in the control group
	(subgroup clinical weakness)) M-A of 10 studies	2018_Yang_1	OR 2.06 95% CI (1.27–3.33), *p* = 0.003; *I*^2^ = 60.6%, *p* = 0.013		Incidence ICUAW: 39% in the corticosteroid group and 23% in the control group. Significant association with a random effects model considering the observed heterogeneity
	(subgroup abnormal EMG) M-A of 10 studies	2018_Yang_1	OR 1.65; 95% CI (0.92–2.95) *p* = 0.093; *I*^2^ = 70.6%, *p* < 0.001		No significant association between corticosteroid use and abnormal electrophysiology (event rate: 46% in both groups)
	Corticosteroids without MV (subgroup) / M-A of 6 studies	2018_Yang_1	OR 1.61 95% CI (0.83–3.13) *p* = 0.161; *I*^2^ = 74.4% *p* = 0.61		Event rate in the corticosteroid group of 31% versus 26% in the control group. No significant association considering the observed heterogeneity
	Use of corticosteroids / M-A of 3 studies	2017_Annoni	OR 2.17 95% CI (1.21–3.91); *I*^2^ = 45%, *p* = 0.16		Use of corticosteroids showed a positive association with ICUAW
	Corticosteroid treatment / 5 individual studies	2017_Sanchéz-Solana	Not M-A		Corticosteroid treatment and CIPNM shows conflicting findings, with most reporting higher CIPNM incidence, one showing an inverse relationship, but no statistically significant association observed
Use of aminoglycosides [21, 23, 29, 36]	Aminoglycoside use / M-A of 3 studies	2022_Yang_Zi	OR 2.51 95% CI (1.54–4.08); *I*^2^ = 0% *p* = 0.41		A significant association was demonstrated between use aminoglycoside and ICUAW
	Aminoglycoside use / M-A of 10 studies	2020_Yang_3	OR 2.06; 95% IC (1.33–3.21) *p* = 0.016; *I*^2^ = 55.7%		The overall effect sizes of the studies revealed a statistically significant relationship between aminoglycoside use and ICUAW, and not to studies limited to patients with abnormal electrophysiology, statistical heterogeneity was obvious
	(subgroup abnormal electrophysiology) / M-A of 7 studies	2020_Yang_3	OR 1.78; 95% CI (0.94–3.39) *p* = 0.08; *I*^2^ = 58.4%, *p* = 0.025		Seven studies assessed the relationship between aminoglycoside use and abnormal electrophysiology, revealing an incidence of 44% in the aminoglycoside group compared to 39% in the control group. However, the overall effect size did not demonstrate a significant association
	(subgroup clinical weakness) / M-A of 3 studies	2020_Yang_3	OR 2.74; 95% CI (1.83–4.10) *p* < 0.01; *I*^2^ = 0% *p* = 0.95		Subgroup and sensitivity analyses indicated a significant association between aminoglycoside use and clinical weakness in specific patient populations. Three studies reported an event rate of 46% in the aminoglycoside group compared to 27% in the control group
	Aminoglycoside use / M-A of 3 studies	2018_Yang_2	OR 2.27; 95% CI (1.07–4.81) *p* < 0.05; *I*^2^ = 69.5% *p* = 0.038		Effect size analysis indicated a statistically significant association between the use of aminoglycosides with ICUAW
	Aminoglycoside use and SIRS / 3 individual studies	2006_Hohl	Not M-A		No significant differences were found regarding particular drugs and the onset of CIPNM. One prospective study SIRS and the use of aminoglycosides were significantly related (*p* = 0.03)
Renal replacement therapy [23, 29]	M-A of 4 studies	2022_Yang_Zi	OR 1.59, 95% CI (1.11–2.28) *p* = 0.01; *I*^2^ = 0%, *p* = 0.60		The combined effect from four studies showed a statistically significant association with good literature consistency
	M-A of 4 studies	2018_Yang_2	OR 0.36 95% CI (0.02–7.05) *p* > 0.05; *I*^2^ = 95.2% *p* < 0.001		There was no effect of RRT on increasing the incidence of ICUAW
Use of norepinephrine (NA) [23]	Days of treatment with NA / M-A of 2 studies	2018_Yang_2	OR 1.04; 95% CI (0.99–1.09) *p* > 0.05; *I*^2^ = 34.2% *p* = 0.218		The overall effect size on the association between days of treatment with NA and ICUAW calculated from 2 studies revealed no significant association
	Treatment with NA / 1 study	2018_Yang_2	Not M-A	HR 1.30; 95% CI (1.08–1.57) *p* < 0.05	Treatment with norepinephrine was found to be a significant risk for developing ICUAW in single study on multivariable analysis
Nutrition [23, 35]	Parenteral nutrition / 1 study	2018_Yang_2	Not M-A	OR 5.11 95% CI (1.14–22.88) *p* = 0.02	Results of a multivariate analysis of a single independent study indicate an association with increased odds of developing ICUAW
	Energy and/or protein delivery / 6 individual studies	2018_Lambell	Not M-A		A variety of methods were used to assess skeletal muscle mass or TBP. Participants in included studies experienced differing levels of muscle loss (0–22.5%) during the first 2 weeks of ICU admission. No association between energy and protein delivery and changes in skeletal muscle mass were observed. Limited evidence exists regarding this association

*ID* identification, *I*^*2*^ Heterogeneity, *N/R* not reported, *SR* Systematic review, *M-A* meta-analysis, *APACHE II* Acute Physiology and Chronic Health disease Classification System II, *ICU* Intensive care unit, *ICUAW* intensive care unit-acquired weakness, *MV* Mechanical Ventilation, *6MWT* Six minute walk test, *CIPNM* Patients critical illness polyneuromyopathy, CIP critical illness polyneuropathy, *SIRS* systemic inflammatory response syndrome, *PCS* prospective cohort studies, *GCS* Glasgow coma scale, *SOFA* Sepsis related organ failure assessment, *MOF* multiple organ failure *LOS* length of stay, *RRT* renal replacement therapy, *TBP* total body protein, *EMG* electromyography, *NCS* nerve conduction studies, *IIT* Intensive insulin therapy, *HBG* high blood glucose, *MRC* Medical Research Council weakness scale, *ICU LOS* Intensive Care Unit Length of stay. *RoB* Risk of Bias, *RCT* Randomized Controlled Trial, *NMBAs* Neuromuscular blocking agents, *RASS* Richmond Agitation-Sedation Scale, *NA* norepinephrine

### Nonmodifiable factors

**Fig. 5:**
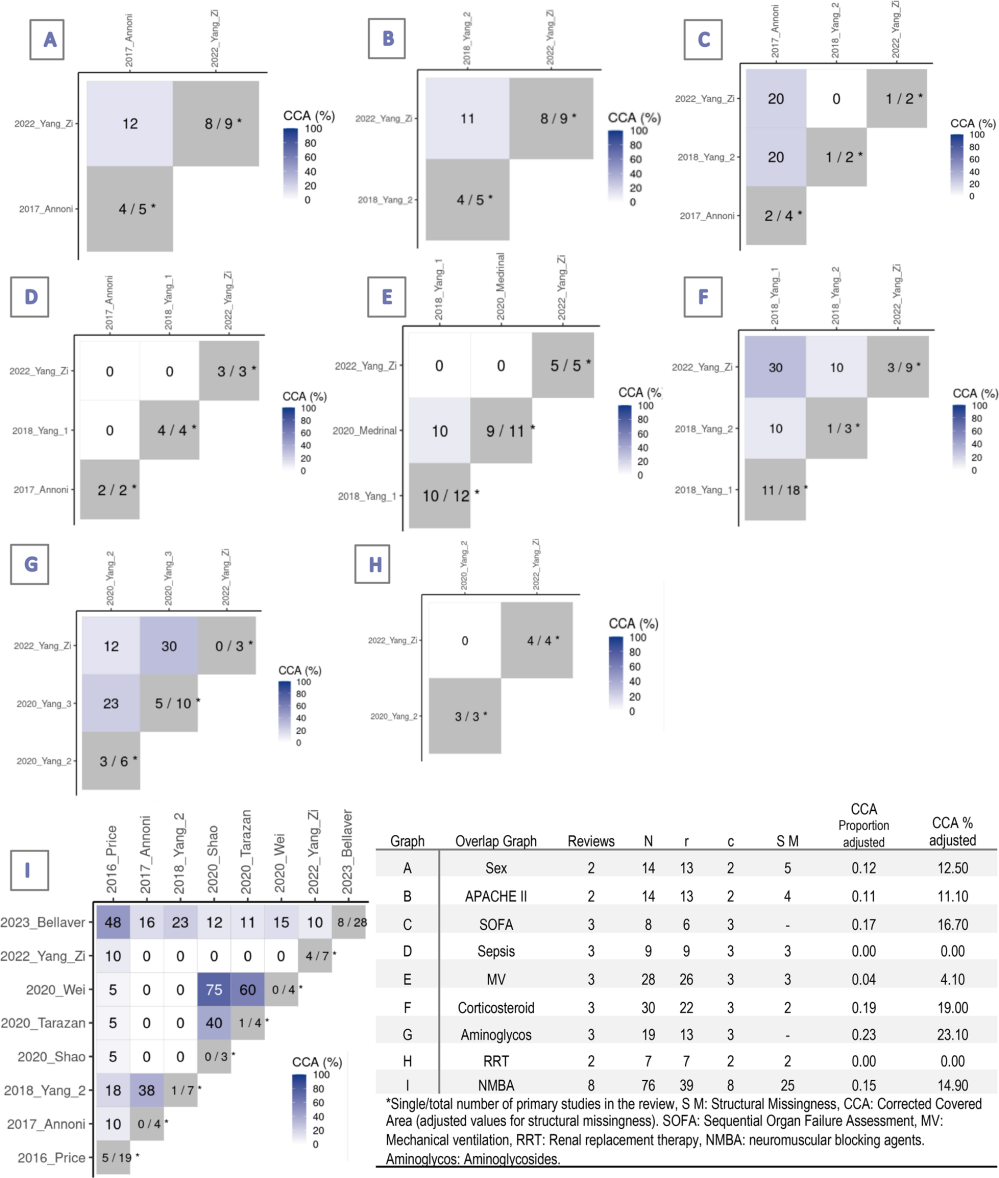
Overlap graph of systematic reviews with meta-analysis for each analyzed outcome. Heatmap, and correlation covered areas (CCA)

Three systematic reviews assessed inherent and pre-existing patient characteristics [23, 29, 39], including biological sex, age, presence of comorbidities (Table 3). The association of ICUAW with biological sex was analyzed in three systematic reviews, including two meta-analyses [29, 39]. The meta-analyses demonstrated association between female biological sex and ICUAW with low heterogeneity. The CCA index for the two reviews was 12.50, indicating high overlap (Fig. 5A). A systematic review showed that the female biological sex was associated with a higher risk of developing ICUAW [23].

The association of age with ICUAW was described in three systematic reviews. A meta-analysis showed a significant association between older age and ICUAW with moderate heterogeneity [29]. Annoni et al. reported a positive association in 4 out of 19 studies analyzed, and their meta-analyses revealed association of age with ICUAW with low heterogeneity [39]. Yang et al. found no association between age and ICUAW with high heterogeneity [23].

One review addressed the presence of pre-existing comorbidities. In the review by Annoni et al. no association between a history of diabetes and ICUAW was detected in the meta-analyses or in the seven other single studies analyzed in the review [39]. No other systematic reviews addressed comorbidities as a risk factor for ICUAW.

Three reviews with meta-analyses detected association between illness severity (APACHE II) and ICUAW but with high heterogeneity between primary studies [23, 29, 39]. APACHE II scores predicted CIPNM in mechanically ventilated patients in another study [36].

No meta-analyses of systematic reviews addressed the association of systemic inflammatory response syndrome (SIRS) with ICUAW. However, Yang et al. [23] described two independent studies showing association between SIRS and ICUAW wherein prolonged SIRS was a risk factor for ICUAW. The review by Hohl et al. [36] concluded that SIRS was a significant predictor of CIPNM based on a primary study.

Four reviews reported results concerning the association between sepsis and ICUAW. One review detected a positive association with low heterogeneity [39]. A more recent review showed an association but was not significant and the heterogeneity was high with no changes after sensitivity analysis [29]. Another review with high heterogeneity showed no association between sepsis and ICUAW [22]. Finally, one review expressed a positive association between sepsis and the duration of sepsis and ICUAW based on a primary study [23]. No overlap was detected (Fig. 5D).

Organ failure, assessed by the sequential organ failure assessment (SOFA) or MOF, was investigated in four reviews with three meta-analyses. Yang et al. [29] and Annoni et al. [39] detected association between SOFA and ICUAW. Although Yang et al. did not detect association between SOFA and ICUAW in their meta-analyses, the results of individual studies suggests that a SOFA score of > 7 and a total SOFA score of > 45 during the 1st week were independent risk factors for ICUAW. Yang et al. also indicated that the duration of dysfunction in two organs and neurological failure were associated with ICUAW in individual studies [23]. The overlap analysis between the systematic reviews was slight (CCA = 0.17) (Fig. 5C).

The association between shock and ICUAW was analyzed in one systematic review. Although meta-analysis was not conducted, the primary study results indicated an association between shock and ICUAW [23]. Infectious diseases were analyzed in one review with a meta-analysis, showing association between infectious diseases and ICUAW with low heterogeneity [29]. Neurological condition or failure (Glasgow coma scale score < 10) was evaluated in a single review without meta-analysis; one primary study detected association between neurological failure and ICUAW [23].

The relationship between MV and ICUAW was analyzed in ten systematic reviews, including four meta-analyses. Yang et al. detected a significant association between MV and ICUAW, but with high heterogeneity [29]. Medrinal et al. detected an association between muscle weakness and a longer duration of MV and ICU stay [28]. Yang et al. reported association between MV with the use of corticosteroids and ICUAW [22]. Annoni et al. detected a positive association between ICUAW and the duration of MV but with high heterogeneity [39]. Hohl et al. indicated that the likelihood of developing CIPNM within 30 days of MV ranged from 8% in the low-risk group to 72% in the high-risk group [36]. Finally, De Jonghe et al. reported that 76% of patients ventilated for > 5 days developed electrophysiological abnormalities, a longer duration of MV, and a twofold increase in mortality [38].

The association between the duration of ICU stay and ICUAW was analyzed in four systematic reviews, including one meta-analysis. The meta-analysis demonstrated a positive association between ICU length of stay and ICUAW, but the heterogeneity between primary studies finding was high even after sensitivity analysis [29].

Only one systematic review [23] analyzed high lactate levels, hyperosmolarity, and electrolyte imbalances, all of which were associated with higher odds of ICUAW in independent studies.

The relationship between severe burns and polyneuropathy was analyzed by McKittrick et al. The occurrence of critical polyneuropathy was detected in 4.4% of the entire burn patient population. And these patients were more likely to have a prolonged ICU stay, which increased the risk of developing CIP [34].

Respiratory muscle dysfunction was analyzed by Prentice et al. [33], respiratory muscle dysfunction was less severe compared to peripheral muscles. However, patients with low maximal inspiratory pressure (MIP) and maximal expiratory pressure (MEP) had lower MRC scores and delayed extubation.

### Modifiable factors

Seven modifiable factors related to therapeutic or pharmacological measures used in the treatment of critical illness were evaluated for their association with the development of ICUAW or similar conditions (Table 4).

In the meta-analysis conducted by Yang et al., it was found that blood glucose levels were correlated with ICUAW. Notably, this association was observed without heterogeneity in the results after large-scale studies were excluded from the analysis [29]. No association between glucose and ICUAW was observed by Annoni et al., although the meta-analyses exhibited high heterogeneity [39]. Four reviews described independent studies; one highlighted the association of hyperglycemia with higher ICUAW risk [23]. Another systematic review described a reduction in CIPNM in patients with glycemic control using insulin in combination with early mobilization [32]. Other reviews detected a reduction in the risk and incidence of CIPNM with intensive glycemic control therapy and strict glucose control (< 6.1 mmol/L) [31, 36].

The relationship between NMBAs and ICUAW was the focus of most meta-analyses but the results were contradictory. Five meta-analyses showed no association between NMBAs and ICUAW [24–26, 29, 39]. Only one meta-analysis detected association between NMBAs and ICUAW; although the analysis exhibited low heterogeneity, possible bias due to small studies with significant associations could have influenced [27]. In the other meta-analyses, high heterogeneity was notable no conclusive associations [23, 30]. The overlap was high (CCA = 14.9%, Fig. 5I).

Contradictory relationships between corticosteroid treatment and ICUAW were reported in five systematic reviews. One meta-analyses of 18 studies demonstrated a significant association between corticosteroids and a higher risk of ICUAW, with a 67.2% of heterogeneity. Subgroup analyses also yielded conflicting results, based on clinical evaluation results, an association between corticosteroid treatment and ICUAW was detected; however, no association was detected based on electrophysiological analysis [22]. Annoni et al. in a meta-analyses of 3 studies showed a positive association with ICUAW with moderate heterogeneity [39]. Others meta-analyses found no significant association with significant heterogeneity [23, 29]. The CCA for the meta-analyses was very high, 19% (Fig. 5F). The other review did not provide definite findings [32].

Analyses of the relationship between aminoglycosides and ICUAW yielded mixed results. The most recent systematic review by Zi Yang et al. [29] detected association between aminoglycosides and ICUAW with no heterogeneity, whereas two reviews by Tao Yang et al. found association between aminoglycosides and ICUAW with significant heterogeneity [21, 23]. It is important to highlight that when examining patient subgroups, including clinical and electrophysiological evaluations, it was found that aminoglycosides showed no significant association with ICUAW in the electrophysiology subgroup, unlike the clinically evaluated subgroup [21]. The CCA for the meta-analyses was very high (CCA = 23.10%) (Fig. 5G). Finally, Hohl et al. [36] detected association between aminoglycoside use and ICUAW in only one prospective study in patients with SIRS.

Two meta-analyses were conducted on the association between RRT and ICUAW. The systematic review by Zi Yang et al. included four studies and showed association between RRT and ICUAW with good consistency in findings [29]. However, the meta-analysis by Tao Yang et al. reported no association between RRT and ICUAW, with high heterogeneity [22]. No overlap was detected (Fig. 5H).

Only one review focused on the association of norepinephrine use and ICUAW. The meta-analysis with low heterogeneity showed no association between norepinephrine use and ICUAW. However, in the same review, an individual study revealed that norepinephrine treatment was associated with ICUAW in a multivariable analysis [23].

Only two reviews analyzed the association between nutritional support and ICUAW. Yang et al. reported one study showing a significant association between nutritional support and ICUAW based on a multivariable analysis [22]. Lambell et al. [35] evaluated the effects of energy and/or protein delivery in six studies using various methodologies to assess skeletal muscle mass or the cross-sectional area of the biceps. In this review muscle loss ranged from 0 to 22.5% during the first 2 weeks of ICU admission and no association was detected between the delivery of energy and proteins and these changes in skeletal muscle mass.

## Discussion

The risk factors identified were scattered across various systematic reviews, none of which comprehensively covered all the risk factors or employed a definitive categorization. In this context, categorizing the risk factors as modifiable and nonmodifiable is based on established definitions in the literature, as highlighted in a narrative review by experts in ICUAW [3]. This categorization not only enhances the analysis but also amplifies the clinical utility of the findings. Distinguishing between factors that clinicians can modify and those that are immutable allows for more targeted and effective patient management in the ICU.

Of the nonmodifiable factors, age, female biological sex, and MOF, which were included in most of the reviews, were consistently associated with a higher risk of developing ICUAW. Advanced age may be related to decreased physiological reserve and increased vulnerability to complications [40, 41]. However, the review by Rooij et al. concluded that the risk of loss of functionality during an ICU stay is not solely dependent on advanced age but is also influenced by the patient's prior state, both cognitively and functionally [37]. One study focused on skeletal muscle metabolism in the context of ICUAW detected sex-specific differences in muscle strength, insulin sensitivity, muscle metabolites, protein degradation pathways, and the cross-sectional area of myocytes. These findings complement the analysis showing that females may be at a disadvantage in the context of ICUAW [42]. MOF, indicating more severe and prolonged illness, was identified as a risk factor for ICUAW in others reviews too [2, 3, 43]. Although MV, disease severity upon ICU admission, and sepsis were the most studied factors, they showed greater heterogeneity in the meta-analyses. This may be due to the diverse pathologies and the severity and type of illness, each with different recovery times, in patients admitted to the ICU [44].

The ICU length of stay, infectious diseases, and the presence of comorbidities may be associated with ICUAW, but these associations cannot be confirmed due to the high heterogeneity between primary studies in reviews, despite the meta-analysis showing an association. No meta-analyses were available for SIRS, neurological failure, shock, high lactate levels, hyperosmolarity, severe burns, or respiratory muscle dysfunction. Conclusions could not be drawn based on primary study descriptions only. However, the potential risk of high lactate levels cannot be overlooked. Lactate is the main metabolite of anaerobic glycolysis induced by hypoperfusion and tissue hypoxia. Hypoperfusion and hypoxia can cause muscle damage and mitochondrial dysfunction, contributing to the onset of ICUAW. Lactate can also act as an inflammatory and oxidative mediator that can contribute to ICUAW [45]. However, more specific studies are needed.

We identified a significant gap concerning the relationship between intrinsic and pre-existing characteristics of critically ill patients and ICUAW. Among these factors, high BMIs or obesity [46–50], prior frailty [40, 41, 51], comorbidities concurrent with the baseline condition or specific pathologies that triggered admission to the ICU including previous strokes, kidney dysfunction, decreased cardiac function, chronic pulmonary disease [44], cardiac surgery [52, 53], severe COVID-19 [54, 55], may play an important role as additional risk factors for ICUAW.

Of note, we did not find systematic reviews that specifically analyze the relationship between obesity and ICUAW. Whether obesity is a risk or protective factor is still under debate. An “obesity paradigm” has been proposed, hypothesizing that obese patients might be able to metabolize their excessive adipose reserves as a predominant energy source and preserve muscle mass during critical illness [56]. However, a study in critically ill patients suggests that obese and nonobese individuals experience muscle mass loss in a similar fashion [46]. Additionally, “sarcopenic obesity” has been proposed, in which fat accumulation and muscle mass loss mutually influence each other, resulting in muscles with excess fat [47]. Obesity also affects calcium signaling and proteins like adiponectin and actinin, influencing muscle contraction [48]. Furthermore, obesity may cause low-grade chronic inflammation, characterized by elevated levels of proinflammatory cytokines and adipokines during critical illness (i.e., an exacerbated inflammatory response in obese patients), which could increase the risk of muscular complications, including ICUAW. Zhao et al. [49] and Hogue et al. [50] investigated the relationship between mortality, MV, and hospital stay in critically ill obese patients but did not address functional outcomes. Both investigations showed that obesity did not increase mortality but did prolong MV, which may impact the incidence of ICUAW. Both reviews highlight the need for further research.

Frailty is a multidimensional syndrome characterized by a decrease in physiological and adaptive reserves, increasing vulnerability to adverse events. Frailty may be an important risk factor for the development of ICUAW. Preliminary epidemiological data suggest a high prevalence of frailty among critically ill patients, which may increase due to the demographic transition of the population [51]. In a systematic review and meta-analysis, Muscedere et al. [41] showed that frailty at the time of ICU admission impacts in hospital and long-term mortality. Additionally, frail patients are less likely to be discharged to return to their homes. Although Muscedere et al. did not address outcomes associated with physical function, this review highlights the potential use of frailty as an independent prognostic predictor in critically ill patients. However, a current systematic review aimed at assessing the impact of age, frailty, and comorbidities on ICU outcomes concluded that these variables were not evaluated in RCTs [57].

Results concerning the association between modifiable factors and ICUAW were inconsistent, reflecting the complex interplay of various therapeutic interventions. Critical factors, including drug dosage, timing of administration, duration of drug usage, and specific pathology being treated, underscore the nuanced impact of these variables on patient outcomes [2, 58, 59].

All reviews concerning the use of aminoglycosides showed significant associations between ICUAW and aminoglycoside use, but half of the meta-analysis exhibited high heterogeneity. Aminoglycosides affect neuromuscular transmission and neurotoxicity and may be involved in the development of ICUAW. Despite the lack of evidence, experts recommend careful monitoring of aminoglycoside levels in the blood and appropriate dosing [2, 60].

Although the results for hyperglycemia were contradictory, glucose variability should be considered in the prevention and treatment of myopathies in critically ill patients [2, 61, 62]. Establishing standards for glycemic control (between 90 and 144 mg/dl) [60] and using intensive insulin therapy may reduce ICUAW [62].

Meta-analyses have produced conflicting results regarding the association between NMBAs and neuromuscular complications. Some studies suggest that NMBAs are not significantly associated with muscle weakness when used alone. However, concurrent use of NMBAs and corticosteroids may elevate the risk of muscle weakness [59]. It is noteworthy that the administration of neuromuscular blockers may affect muscle nerve excitability, potentially leading to muscle weakness in critically ill patients. This interaction with neuromuscular function could pose a risk factor for the development of ICUAW, particularly when combined with other factors such as the duration of mechanical ventilation and illness severity. It is critical to acknowledge that factors like the duration of NMBA infusion, specific patient demographics (e.g., septic patients with multiorgan dysfunction), and simultaneous corticosteroid use might modify the risk associated with NMBAs. Furthermore, some NMBA compounds may share structural similarities with steroids, possibly intensifying the risk of developing myopathies. In summary, while NMBAs may not independently constitute a risk factor for ICUAW in most cases, their use in conjunction with factors such as corticosteroids and extended infusion periods might contribute to neuromuscular complications in critically ill patients [58, 59, 63].

Corticosteroids are commonly used in intensive care units and have been linked to ICUAW, despite the lack of consistent results in meta-analyses [22, 23, 29, 39]. However, excessive administration of corticosteroids can cause muscle dysfunction and nerve damage, promote the breakdown of muscle proteins, and increase protein loss. They can also have side effects such as lipodystrophy, and their use may increase the absorption and turnover of fatty acids in adipose tissue, which is closely related to the onset of ICUAW [59, 60, 63–65].

In relation to RRT and acute kidney injury (AKI), a recent literature review highlights the pathophysiological mechanisms, such as protein degradation, inflammation, and metabolic pathway alterations, through which AKI and its treatment with RRT–AKI may contribute to muscle loss, suggesting a relationship with ICU-AW. Preclinical and clinical data indicate that both AKI and RRT–AKI could influence the development of ICU-AW [66].

Norepinephrine is used in the ICU as a vasoconstrictor and positive inotropic agent to manage shock and sepsis, thereby improving arterial perfusion and pressure. Only one systematic review addressed its use as a potential factor in the development of ICUAW. Primary studies indicate that norepinephrine is significantly associated with an increased risk of ICUAW, with a dose-dependent effect that increases risk with each cumulative dose. Therefore, it is recommended to limit norepinephrine exposure and shorten its administration in clinical practice to reduce the incidence of ICUAW [45, 67].

A single review has demonstrated an association between nutritional intake and ICUAW [35]. Malnutrition and nutritional imbalance may increase the risk of ICUAW. Interestingly, the timing of total parenteral nutrition (TPN) administration appears to influence risk; early TPN may increase the likelihood, while early caloric restriction and delayed TPN administration may mitigate it [3]. It is important to mention that recent research findings indicate that early mobilization combined with timely nutrition support significantly reduced the incidence of ICUAW compared to early mobilization alone or standard care [68].

The impact of other medical treatments, such as the use of Propofol [69] or prolonged use of extracorporeal membrane oxygenation ECMO [70], also may contribute to ICUAW and should be more investigated.

Strategic interventions and proactive monitoring for modifiable risk factors: effective management of modifiable risk factors such as hyperglycemia, neuromuscular blockade, corticosteroids, aminoglycosides, and nutritional support is crucial for minimizing ICUAW risks. Implementing systematic glycemic control strategies tailored to individual patient conditions and refining guidelines for neuromuscular blocking agents are essential to balance benefits against the risks of prolonged use. Additionally, precise protocols for the timing and dosage of aminoglycosides require frequent monitoring to prevent ICUAW while effectively treating underlying conditions. Early detection and consistent monitoring enable clinicians to tailor interventions that mitigate risks and improve outcomes, necessitating regular evaluation of drug dosages, treatment timing, and ongoing patient conditions to adjust treatment protocols effectively.

The early detection and consistent monitoring of modifiable risk factors are critical for preventing and managing ICUAW. This proactive approach enables clinicians to tailor interventions that mitigate risk and improve patient outcomes. Regular evaluation of variables such as drug dosages, treatment timing, and ongoing patient conditions is essential for adjusting treatment protocols and ensuring effective management of ICUAW.

In an effort to identify patients at risk of developing ICUAW, various predictive models have been developed [52, 60, 64, 65] A recent systematic review by Zhang et al. [63], identified 11 risk models for ICUAW. These models incorporate a variety of predictors based on the type of diseases of the participants, conceptual definitions, and diagnostic tools used. Additionally, some studies have incorporated more specific variables, such as electrodiagnostic tests and ultrasound of the quadriceps rectus femoris muscle (QRF).

The evaluation of these models shows that their values in the area under the receiver operating characteristic curve (ROC) range from 0.7 to 0.923, indicating a moderate to high discriminatory capacity between patients with and without ICUAW. However, it is noted that most of the models analyzed exhibit certain biases, such as lack of blinding, incomplete reporting, insufficient sample sizes, lack of external validation, and inadequate calibration of the models. Therefore, it is concluded that although some models prove effective in predicting ICUAW, it is crucial to address these deficiencies and conduct additional studies to validate and refine the accuracy of these predictive models before their widespread implementation in clinical settings.

Our findings suggest that predictive models for ICUAW should be flexible and incorporate both modifiable and nonmodifiable factors associated with the condition. It is vital to consider factors that have demonstrated a consistent association, such as age, female gender, and organ failure. Additionally, it is essential to account for factors that may not have a conclusive association due to heterogeneity found in systematic reviews or their absence, yet have a significant pathophysiological basis in the development of ICUAW as discussed in this text. Prominent among these factors are comorbidities such as obesity, frailty, high lactate levels, hyperglycemia, the use of NMBAs, corticosteroids, aminoglycosides, renal replacement therapy, norepinephrine, and nutritional intake.

### Strengths and limitations

The findings of this review are primarily based on individual reviews. Any biases, methodological errors, or limitations present in the original reviews could impact the conclusions. The associations identified in this study may be affected by the heterogeneity highlighted in the meta-analyses and the lack of meta-analyses for some factors. We only reported the extent of overlap among the meta-analyses and did not devise a strategy to resolve this aspect. Nevertheless, we generated the overlap analysis matrices and a map delineating the primary studies incorporated in each systematic review (Supplementary Material), which can be utilized for subsequent in-depth analyses.

It is important to note that this review highlighted factors described in selected systematic reviews, leading to limited discussion of other potential factors not addressed in those reviews. Many of these unaddressed factors are related to therapies performed in the ICU, whose causal relationships remain unclear. However, the discussion briefly mentions conclusions from primary studies and narrative reviews which emphasize their possible implications with ICUAW.

A comprehensive literature search was conducted using a sensitive approach to identify all relevant reviews related to ICUAW. Nevertheless, the search may be limited by the omission of other databases or publications in nonconventional languages (primarily Asian languages), which could result in the absence of relevant reviews.

## Conclusions

This overview identifies nonmodifiable risk factors for ICU-acquired weakness, such as advanced age, female sex, and organ failure, with the need for targeted monitoring in these patient groups. While modifiable factors like glucose control, neuromuscular blockade, corticosteroid use, aminoglycosides, renal replacement therapy, and norepinephrine show variable impacts on ICUAW risk, it is important to note that some risk factors have yielded contradictory results and high heterogeneity. Furthermore, certain factors remain under-researched, highlighting a persistent need for studies with a more personalized focus that encompass all potential factors contributing to the development of ICUAW. The development of preventive approaches tailored to the complexities of ICUAW is also essential. Our findings underline the necessity of individualized treatment strategies to enhance patient outcomes in the ICU.

## Supplementary Information

Supplementary Material 1.

Supplementary Material 2.

## Data Availability

The datasets used and/or analyzed during the current study are available from the corresponding author on reasonable request. The detailed data extracted from the systematic reviews supporting the conclusions of this article are included in the supplementary material. An additional table with extended data is available at the following link: https://l1nk.dev/ZxMLC

## Abbreviations

ICUAW: Intensive Care Unit-Acquired Weakness
MRC: Medical Research Council
CIPNM: Critical illness polyneuromyopathy
CIP: Critical illness polyneuropathy
CIM: Critical illness myopathy
PROSPERO: International Prospective Register of Systematic Reviews
JBI: Joanna Briggs Institute
PRIOR: Preferred reporting items for overviews of reviews
PRISMA: Preferred reporting items for systematic reviews and meta-analyses
CCA: Corrected covered area
ccaR: R package used for overlap analyses
ROBIS: Risk of bias in systematic reviews
6MWT: 6-Minute walk test
APACHE II: Acute physiology and chronic health evaluation II
SIRS: Systemic inflammatory response syndrome
SOFA: Sequential organ failure assessment
MOF: Multiple organ failure
MV: Mechanical ventilation
MIP: Maximal inspiratory pressure
MEP: Maximal expiratory pressure
RRT: Renal replacement therapy
AKI: Acute kidney injury
TPN: Total parenteral nutrition
ECMO: Extracorporeal membrane oxygenation
QRF: Quadriceps rectus femoris
ROC: Receiver operating characteristic
