# Supplementary material for: Which factors are associated with acquired weakness in the ICU? An overview of systematic reviews and meta-analyses
Source: J Intensive Care. 2024 Sep 5;12:33. doi: 10.1186/s40560-024-00744-0 (PMC11375885; doi:10.1186/s40560-024-00744-0)
Supplement: Supplementary file 1 — Supplementary Material 1. [file 40560_2024_744_MOESM1_ESM.pdf]

Supplementary Material Manuscript:

Risk factors for intensive care unit-acquired weakness: an overview of systematic reviews and meta-analyses

METHODS

An overview of systematic reviews was conducted. The protocol was registered in PROSPERO (CDR-REGISTER 207863), and the guidelines set forth by the JBI Collaboration (1) Aromataris et al. (2) and the PRIOR statement (3) were followed.

Search strategy

A systematic, sensitive, and reproducible search strategy was conducted. The strategy was updated until August 2023. The following electronic databases were searched: MEDLINE, EMBASE, CINAHL, Cochrane Library, Google Scholar, and Epistemonikos. The complete search strategies are shown in Supplementary Table 1.

Selection criteria

Systematic reviews with or without meta-analyses were included. Only reviews with critical methodological components (3), such as exhaustive search and risk of bias assessment, were included in the analysis. The target population was adult patients hospitalized in critical care units. Risk factors (exposure) included any conditions evaluated as a risk for the development of ICUAW or functional deterioration, such as pre-existing factors or comorbidities, conditions at ICU admission, disease progression, stages of illness, and medical or pharmacological treatments. Reviews that considered

ICUAW or similar diagnoses, such as critical illness polyneuromyopathy (CIPNM), critical illness polyneuropathy (CIP), and critical illness myopathy (CIM), and any outcomes related to muscular weakness or physical functioning, such as mobility tests or performance in activities, were included. No language or date restrictions were applied.

Study selection and data extraction

The search results were aggregated in COVIDENCE® (4) to identify duplicates and select studies according to the previously described criteria. The screening was independently conducted by two pairs of collaborators (RGA, RTC, GMN, FGS). Discrepancies were resolved via the intervention of a third reviewer (RFA) and a senior researcher (PS). The selection process and reasons for exclusion are presented in accordance with the PRISMA 2020 flow diagram (5). A standardized data collection form was developed. Data extraction carried out by the lead author (RFA) was verified by two coauthors (RGA, GMN). Discrepancies were resolved by a senior researcher (PS). The authors of the eligible studies were contacted to obtain missing data.

The characteristics extracted from the selected systematic review included: year, author, title, type of study (systematic review with or without meta-analyses), population characteristics, study designs, number of primary studies, the range of participants included in the reviews, and the diagnostic tool. The number of primary studies meeting the purpose of this review is reported. The quantitative and descriptive reports related to the objective of this overview and the conclusions of the systematic reviews concerning specific outcomes were extracted.

Risk of bias assessment

The risk of bias for the included systematic reviews was assessed using the ROBIS tool, which is recommended for it (6). Two reviewers (GMN, RGA) independently assessed the risk of bias and any disagreements were resolved by the senior investigator (PS) and consensus with the principal researcher (RFA). Risk of bias was not a criterion for study eligibility or exclusion in this overview. A graphical representation of the bias risks was created using the templates from the "resources for ROBIS tool" at the University of Bristol (7).

Data analysis

The risk factor results are summarized qualitatively, presented in tables, and organized into nonmodifiable factors and modifiable factors similar to previous studies (8) (9). Nonmodifiable factors included pre-existing conditions, baseline characteristics upon ICU admission, comorbidities, and natural disease progression. Modifiable factors included medical and pharmacological interventions during the ICU stay. The characteristics of each study and the combined results are presented descriptively through tables. The corrected covered area (CCA) index was calculated to assess the degree of overlap in primary studies between the systematic reviews and meta-analyses. A heatmap was generated using the ccaR package (10), and the corresponding analyses of overlap are provided (11).

References

1. Aromataris E, Munn Z. JBI Manual for evidence synthesis. JBI, editor2021.
2. Aromataris E, Fernandez R, Godfrey CM, Holly C, Khalil H, Tungpunkom P. Summarizing systematic reviews: methodological development, conduct and reporting of an umbrella review approach. Int J Evid Based Healthc. 2015;13(3):132-40.
3. Gates M, Gates A, Pieper D, Fernandes RM, Tricco AC, Moher D, et al. Reporting guideline for overviews of reviews of healthcare interventions: development of the PRIOR statement. BMJ. 2022;378:e070849.
4. Covidence systematic review software Melbourne, Australia. [Available from: Available at www.covidence.org.
5. Haddaway NR, Page MJ, Pritchard CC, McGuinness LA. PRISMA2020: An R package and Shiny app for producing PRISMA 2020-compliant flow diagrams, with interactivity for optimised digital transparency and Open Synthesis. Campbell Syst Rev. 2022;18(2):e1230.
6. Whiting P, Davies P, Savović J, Caldwell D, Churchill R, group R. Evidence to inform the development of ROBIS, a new tool to assess the risk of bias in systematic reviews. Journal of Clinical Epidemiology. 2015.
7. Whiting P. ROBIS tool and resources 2023 [Available from: https://www.bristol.ac.uk/population-health-sciences/projects/robis/resources/
8. Vanhorebeek I, Latronico N, Van den Berghe G. ICU-acquired weakness. Intensive Care Med. 2020;46(4):637-53.
9. Flower L, Puthucheary Z. Muscle wasting in the critically ill patient: how to minimise subsequent disability. Br J Hosp Med (Lond). 2020;81(4):1-9.
10. Bougioukas KI, Diakonidis T, Mavromanoli AC, Haidich AB. ccaR: A package for assessing primary study overlap across systematic reviews in overviews. Res Synth Methods. 2023;14(3):443-54.
11. Pieper D, Antoine SL, Mathes T, Neugebauer EA, Eikermann M. Systematic review finds overlapping reviews were not mentioned in every other overview. J Clin Epidemiol. 2014;67(4):368-75.

Table **1** Structure of a search strategy in data bases

Structure of a search strategy in data bases<br>Date search: last update: 04-08-2023; (preliminary search started: 16-09-2020)		
History and Search Details - MEDLINE actualized el 04-08-2023		
Interface - PUBMED	((("Critical Care"[Mesh] OR "Critical Illness"[Mesh] OR "Intensive Care Unit*"[Mesh] OR "Critical Patient*" OR "critically ill patient*" OR "critical ill patient*" OR "critically ill" OR "critical Illnesses" OR ICU OR "critical care unit" OR "intensive care") AND ("Muscular Atrophy"[Mesh] OR "Muscle Weakness"[Mesh] OR "Atrophy"[Mesh] OR "Activities of Daily Living"[Mesh] OR "Sarcopenia"[Mesh] OR "muscle atrophy" OR "disuse atrophy" OR "skeletal muscle atrophy" OR "skeletal muscle loss" OR "muscle weakness" OR "muscular strength" OR "muscle thickness" OR "loss of muscle mass" OR "weakness" OR "muscle fiber weakness" OR "contractile weakness" OR "muscle injury" OR "muscle deconditioning" OR "muscle degeneration" OR "muscle loss" OR "muscle hypotonia" OR "muscle wasting" OR "physical funct*" OR 'physical strength' OR "strength loss" OR "functional capacity" OR "physical activity" OR "loss of functionality" OR "functional status" OR "physical status" OR "functional loss" OR "inactivity" OR "physical impairments" OR "impairments" OR "physical capabilities" OR "physical abilities" OR "muscular disease*" OR "sarcopenia" OR "sarcopenia elderly" OR "sarcopenia obesity" OR "intensive care unit-acquired weakness" OR icuaw OR "icu-acquired weakness" OR "critical illness polyneuropathy" OR "critical illness myopathy" OR "critical illness neuromyopathy" OR frailty OR "physical frailty" OR "ventilator induced diaphragmatic dysfunction" OR "VIDD" OR "diaphragmatic dysfunction" OR "diaphragm thickness" OR "immobilization" OR "movement" OR "motor dysfunction" OR "less mobile" OR "mobility status" OR disability OR "loss of autonomy" OR "ambulation capacity" OR "motor function" OR "motor activity" OR "impaired quality of life" OR "lower quality of life" OR "decreased quality of life" OR "adl function" OR "daily living function" OR "disability in activities of daily living" OR "activities of daily life"))) AND ((meta-analysis[Publication Type] OR meta analysis[Publication Type] OR meta analysis[Title/Abstract] OR meta analysis[MeSH Terms] OR review[Publication Type] OR search*[Title/Abstract]))	Filters applied: Humans<br><br>Filters SR: AND (meta-analysis[Publication Type] OR meta analysis[Publication Type] OR meta analysis[Title/Abstract] OR meta analysis[MeSH Terms] OR review[Publication Type] OR search*[Title/Abstract])<br><br>RESULTS:<br>3195
History and Search Details - CINAHL actualized el 04-08-2023		
Interface - EBSCOhost Research Databases	("Critical Care"[Mesh] OR "Critical Illness"[Mesh] OR "Intensive Care Unit*"[Mesh] OR "Critical Patient*" OR "critically ill patient*" OR "critical ill patient*" OR "critically ill" OR "critical Illnesses" OR ICU OR "critical care unit")) AND (("Muscular Atrophy"[Mesh] OR "Muscle Weakness"[Mesh] OR "Atrophy"[Mesh] OR "Activities of Daily Living"[Mesh] OR "Sarcopenia"[Mesh] OR "physical funct*" OR "physical strength" OR "functional capacity" OR "physical act*" OR "loss of functionality" OR "functional status" OR "physical status" OR "Physical inactivity" OR "functional loss" OR "inactivity"OR "physical impairments" OR "impairments" OR "physical capabilities" OR "physical abilities" OR "strength loss" OR "muscle hypotonia" OR "muscular disease*" OR "sarcopenia elderly" OR "sarcopenia obesity" OR "Intensive care unit-acquired weakness" OR ICUAW OR "ICU-acquired weakness" OR "muscle weakness" OR "loss of muscle mass" OR "muscle loss" OR "muscle atrophy" OR "muscle degeneration" OR "disuse atrophy" OR "muscle deconditioning" OR "critical illness polyneuropathy" OR "critical illness myopathy" OR "critical illness neuromyopathy" OR Frailty OR "muscle wasting" OR "ventilator-induced diaphragmatic dysfunction" OR "physical frailty" OR frailty OR "physically frail" OR "immobilization" OR "movement" OR "less mobile" OR "mobility status" OR "disabilit*" OR "loss of autonomy" OR "ambulation capacity" OR "motor function" OR "motor activity" OR "impaired quality of life" OR "lower quality of life" OR "decreased quality of life" OR	Limitadores - Excluir registros MEDLINE; Humano<br>Ampliadores - Aplicar palabras relacionadas; Buscar también dentro del texto completo de los artículos<br>Modos de búsqueda - Booleano/Frase<br><br>RESULTS:<br>1130

	"ADL function" OR "daily living function" OR "disability in activities of daily living" OR "activities of daily life")) AND TI ((meta-analysis OR "meta analysis" OR "meta analysis"[MeSH Terms] OR review OR search* OR "systematic review"))	
History and Search Details - EMBASE (09-08-23)		
Interface - Elsevier database	('intensive care'/exp OR 'intensive care' OR 'critical illness'/exp OR 'critical illness' OR 'intensive care unit'/exp OR 'intensive care unit' OR 'critical care'/exp OR 'critical care' OR 'critical patient' OR 'critically ill patient'/exp OR 'critically ill patient' OR 'critically ill'/exp OR 'critically ill' OR 'critical illnesses' OR icu OR 'critical care unit'/exp OR 'critical care unit') AND ('muscular atrophy'/exp OR 'muscular atrophy' OR 'atrophy'/exp OR 'atrophy' OR 'activities of daily living'/exp OR 'activities of daily living' OR 'sarcopenia'/exp OR 'sarcopenia' OR 'physical funct*' OR 'physical strength'/exp OR 'physical strength' OR 'functional capacity'/exp OR 'functional capacity' OR 'physical act*' OR 'loss of functionality' OR 'functional status'/exp OR 'functional status' OR 'physical status'/exp OR 'physical status' OR 'physical inactivity'/exp OR 'physical inactivity' OR 'functional loss' OR 'inactivity'/exp OR 'inactivity' OR 'physical impairments' OR 'impairments' OR 'physical capabilities' OR 'physical abilities' OR 'strength loss' OR 'muscle hypotonia'/exp OR 'muscle hypotonia' OR 'muscular disease*' OR 'sarcopenia elderly' OR 'sarcopenia obesity' OR 'intensive care unit-acquired weakness' OR icuaw OR 'icu-acquired weakness' OR 'muscle weakness'/exp OR 'muscle weakness' OR 'loss of muscle mass' OR 'muscle loss'/exp OR 'muscle loss' OR 'muscle atrophy'/exp OR 'muscle atrophy' OR 'muscle degeneration'/exp OR 'muscle degeneration' OR 'disuse atrophy'/exp OR 'disuse atrophy' OR 'muscle deconditioning' OR 'critical illness polyneuropathy'/exp OR 'critical illness polyneuropathy' OR 'critical illness myopathy'/exp OR 'critical illness myopathy' OR 'critical illness neuromyopathy' OR 'frailty'/exp OR frailty OR 'muscle wasting'/exp OR 'muscle wasting' OR 'ventilator-induced diaphragmatic dysfunction' OR 'physical frailty'/exp OR 'physical frailty' OR 'immobilization'/exp OR 'immobilization' OR 'movement'/exp OR 'movement' OR 'motor dysfunction'/exp OR 'motor dysfunction' OR 'less mobile' OR 'mobility status' OR 'disabilit*' OR 'loss of autonomy' OR 'ambulation capacity' OR 'motor function'/exp OR 'motor function' OR 'motor activity'/exp OR 'motor activity' OR 'impaired quality of life' OR 'lower quality of life' OR 'decreased quality of life' OR 'adl function' OR 'daily living function' OR 'disability in activities of daily living' OR 'activities of daily life') AND ('systematic review'/exp OR 'systematic review':ta OR 'meta analysis'/exp OR 'meta analysis') AND [embase]/lim NOT ([embase]/lim AND [medline]/lim)	('systematic review'/exp OR 'systematic review':ta OR 'meta analysis'/exp OR 'meta analysis')<br>(EMBASE systematic review filters tested)<br>Filtro<br>HUMANOS<br>No MEDLINE<br>RESULTS: 2218
History and Search Details - Cochrane Database 04-08-2023		
Search	ID Search Hits<br>#1 ("Critical Care"):ti,ab,kw (Word variations have been searched) 4339<br>#2 "critical Illnesses" 174<br>#3 "Intensive Care Units" 7225<br>#4 MeSH descriptor: [Intensive Care Units] explode all trees 4100<br>#5 "Critical Patient*" 66<br>#6 "critically ill patient*" 2679<br>#7 "critical ill patient*" 3<br>#8 "critically ill" 8084<br>#9 "medical intensive" 688<br>#10 "critical Illnesses" 174<br>#11 ICU 16161<br>#12 "critical care unit" 335<br>#13 #1 OR #2 OR #3 OR #4 OR #5 OR #6 OR #7 OR #8 OR #9 OR #9 OR #10 OR #11 OR #12 27488<br>#14 MeSH descriptor: [Muscular Atrophy] 1 tree(s) exploded 990	Filters<br>Cochrane Reviews<br><br>Results<br><br>Total 1336<br><br>Cochrane Protocols<br>Total<br>134

	#15 "muscle atrophy" 929	
	#16 "muscle weakness" 3291	
	#17 "atrophy" 7345	
	#18 “disuse atrophy” 82	
	#19 "skeletal muscle atrophy" 55	
	#20 "skeletal muscle loss" 42	
	#21 “muscle weakness” 3291	
	#22 "muscle wasting" 592	
	#23 "muscular strength" 1569	
	#24 "muscle thickness" 888	
	#25 "loss of muscle mass" 392	
	#26 weakness 8284	
	#27 "muscle fiber weakness" 0	
	#28 "contractile weakness" 1	
	#29 "muscle injury" 1491	
	#30 “muscle deconditioning” 36	
	#31 "muscle degeneration" 41	
	#32 “muscle loss” 400	
	#33 “muscle hypotonia” 150	
	#34 "diaphragm atrophy" 13	
	#35 "physical function" 8624	
	#36 "physical strength" 212	
	#37 "strength loss" 134	
	#38 "functional capacity" 4820	
	#39 "physical activity" 38539	
	#40 "loss of functionality" 23	
	#41 "functional status" 9368	
	#42 "physical status" 7470	
	#43 "physical inactivity" 1619	
	#44 "functional loss" 180	
	#45 "inactivity" 2470	
	#46 "physical impairments" 180	
	#47 "impairments" 7221	
	#48 "physical capabilities" 52	
	#49 "physical abilities" 169	
	#50 "muscular disease" 58	
	#51 "sarcopenia" 1925	
	#52 “sarcopenia elderly” 8	
	#53 "sarcopenia obesity" 6	
	#54 "intensive care unit acquired weakness" 57	
	#55 icuaw 74	
	#56 "icu-acquired weakness" 95	
	#57 "critical illness polyneuropathy" 39	
	#58 “critical illness myopathy” 19	
	#59 "critical illness neuromyopathy" 2	
	#60 frailty 2691	
	#61 "physical frailty" 167	
	#62 ”ventilator induced diaphragmatic dysfunction” 14	
	#63 vidd 20	
	#64 "diaphragmatic dysfunction" 62	
	#65 "diaphragm thickness" 87	
	#66 "immobilization" 2935	
	#67 “movement” 35198	
	#68 "motor dysfunction" 2316	
	#69 "less mobile" 22	
	#70 “mobility status” 41	
	#71 disability 42228	
	#72 “loss of autonomy” 81	
	#73 “ambulation capacity” 5	
	#74 “motor function” 6540	

	#75 “motor activity” 5358<br>#76 “impaired quality of life” 580<br>#77 “lower quality of life” 471<br>#78 “decreased quality of life” 608<br>#79 “adl function” 85<br>#80 “daily living function” 75<br>#81 “disability in activities of daily living” 51<br>#82 “activities of daily life” 417<br>#83 “activities of daily living” 12488<br>#84 #14 OR # 15 OR # 16 OR #17 OR #18 OR #19 OR #20 OR #21 OR #22 OR #23 OR #24 OR #25 OR #26 OR #27 OR #28 OR #29 OR #30 OR #31 OR #32 OR #33 OR #34 OR #35 OR #36 OR #37 OR #38 OR #39 OR #40 OR #41 OR #42 OR #43 OR #44 OR #45 OR #46 OR #47 OR #48 OR #49 OR #50 OR #51 OR #52 OR #53 OR #54 OR #55 OR #56 OR #56 OR #57 OR #58 OR #60 OR #61 OR #62 OR #63 OR #64 OR #65 OR #66 OR #67 OR #68 OR #69 OR #70 OR #71 OR #72 OR #73 OR #74 OR #75 OR #76 OR #76 OR #77 OR #78 OR #79 OR #80 OR #81 OR #82 OR #83 608178<br>#85 #13 AND #84 10877	
History and Search Details Epistemónikos database (date search: 09-08-23)		
	"Critical Care" OR "Critical Illness" OR "Intensive Care Unit" OR "Critical Patient" OR "critically ill patient" OR "critical ill patient" OR "critically ill" OR "medical intensive" OR "critical Illnesses" OR ICU OR "critical care unit" AND "Muscular atrophy" OR "muscle atrophy" OR "muscle weakness" OR "atrophy" OR "disuse atrophy" OR "skeletal muscle atrophy" OR "skeletal muscle loss" OR "muscle weakness" OR "muscular strength" OR "muscle thickness" OR "loss of muscle mass" OR "weakness" OR "muscle fiber weakness" OR "contractile weakness" OR "muscle injury" OR "muscle deconditioning" OR "muscle degeneration" OR "muscle loss" OR "muscle hypotonia" OR "physical function" OR 'physical strength' OR "strength loss" OR "functional capacity" OR "physical activity" OR "loss of functionality" OR "functional status" OR "physical status" OR "physical inactivity" OR "functional loss" OR "inactivity" OR "physical impairments" OR "impairments" OR "physical capabilities" OR "physical abilities" OR "muscular disease" OR "sarcopenia" OR "sarcopenia elderly" OR "sarcopenia obesity" OR "intensive care unit-acquired weakness" OR icuaw OR "icu-acquired weakness" OR "critical illness polyneuropathy" OR "critical illness myopathy" OR "critical illness neuromyopathy" OR frailty OR "physical frailty" OR "ventilator induced diaphragmatic dysfunction" OR "VIDD" OR "diaphragmatic dysfunction" OR "diaphragm thickness" OR "immobilization" OR "movement" OR "motor dysfunction" OR "less mobile" OR "mobility status" OR disability OR "loss of autonomy" OR "ambulation capacity" OR "motor function" OR "motor activity" OR "impaired quality of life" OR "lower quality of life" OR "decreased quality of life" OR "adl function" OR "daily living function" OR "disability in activities of daily living" OR "activities of daily life" OR "activities of daily living"	Filters:<br>Publication type: Systematic Review<br>Result:<br>139
Search: Buscador avanzado de Google Scholar – 08-08-23		
	All of the words: "intensive care unit acquired weakness"<br>The exact phrase: “icu acquired weakness”<br>At least one of the words: "systematic review" "meta analysis"<br>Where my words occur: X : in the article<br>Include citations: no<br>Type: reviews<br>Search strategy notes:<br>Due to character limitations in Google Scholar's search bar, the strategy was condensed to the most relevant or characteristic terms of the search query, and terms were included to narrow down to systematic reviews.	Results<br>1253
		9405
