# Supplementary material for: Which factors are associated with acquired weakness in the ICU? An overview of systematic reviews and meta-analyses
Source: J Intensive Care. 2024 Sep 5;12:33. doi: 10.1186/s40560-024-00744-0 (PMC11375885; doi:10.1186/s40560-024-00744-0)
Supplement: Supplementary file 2 — Supplementary Material 2. [file 40560_2024_744_MOESM2_ESM.pdf]

Supplementary Tables and figures:

Table 1 (complementary): Characteristics of the selected studies

ID Article Year_Author	Title	Country of authors' affiliation	Objectives of the included review	Number of databases sourced and searched / date range of database searching	Instrument used to appraise the primary studies	Method of synthesis/analysis employed to synthesize the evidence
2023_Bellaver	Association between neuromuscular blocking agents and the development of intensive care unit-acquired weakness: A systematic review with meta-analysis and trial sequential analysis.	Brazil	To review the literature and synthesize evidence concerning the effects of the use of NMBA regarding the development of intensive care unit-acquired weakness.	PubMed, EMBASE, and Cochrane Central Register of Controlled Trials / to November 2022	ROB of each study using the Newcastle-Ottawa scale for cohort studies and the Cochrane Collaboration tool for RCT	They used OR and MD with 95% CI to express effect estimates for binary and continuous variables. Funnel plot asymmetry detected publication bias. The Cochran Q and I^2 tests assessed study heterogeneity. A random-effects model was used for all analyses. Subgroup analysis explored heterogeneity. Interaction tests and meta-regressions were done to assess subgroup differences. Trial sequential analysis (TSA) evaluated data sufficiency for RCT conclusions.
2022_YangZi	A systematic review and meta-analysis of risk factors for intensive care unit acquired weakness.	China	To evaluate and summarize the risk factors of intensive care unit acquired weakness (ICU-AW).	PubMed, EMBASE, Web of Science, SinoMed, Chinese National Knowledge Infrastructure, Chinese WANFANG, VIP / January 1, 2005 to August 13, 2021	The Newcastle-Ottawa Quality Scale (NOS)	For dichotomous variables, OR with its 95% CI was used. For continuous variables, MD with its 95% CI was used. Initial heterogeneity test assessed. If $p > 0.1$ and $I^2 < 50\%$, data were deemed homogeneous, and fixed-effects model was applied.
2020_Shao	Early neuromuscular blocking agents for adults with acute respiratory distress syndrome: a systematic review, meta-analysis and meta-regression.	China	Secondary outcomes were 21–28 days mortality, NMBA-related complications (ICU-AW, days free of ventilation and days not in the ICU by day 28, MRC score, APACHE II score and arterial oxygen tension	MEDLINE (PubMed), EMBASE, Cochrane Library, WOS and ClinicalTrial.gov databases / August 2020	RoB 1	Summary estimates were generated for categorical variables using RR and 95% CI. Continuous outcomes were summarized using mean and SD. M-A employed M-H random-effect models or fixed-effects models based on heterogeneity. Zero-event trials were adjusted with a correction factor of 1.0. Heterogeneity was assessed using I^2 testing.

			(PaO2)/(FiO2) (at 48 hours and 72 hours).			
2020_Medrinal	ICU outcomes can be predicted by noninvasive muscle evaluation: a meta-analysis.	France	To evaluate the impact of muscle weakness on one or more of the following “critical outcomes.	MEDLINE/Pubmed, EMBASE, CINAHL, Cochrane library, Science Direct / January 2000 to December 2018	QUIPS, QUADAS-2	Random-effects M-A were conducted using DerSimonian & Laird method for binary outcomes. OR and 95% CIs were calculated using the Hartung-Knapp-Sidik Jonkman method.
2020_Tarazan	Neuromuscular blocking agents in acute respiratory distress syndrome: updated systematic review and meta-analysis of randomized trials.	Canada	To determine the efficacy and safety of NMBAs in ARDS, comparing the effects of NMBA as a continuous infusion versus placebo or no NMBA infusion.	MEDLINE (Ovid), EMBASE, Cochrane CENTRAL, Clinical trial registries: ClinicalTrials.gov, ISRCTN Register, and WHO ICTRP / October 2012 to July 2019	RoB 1	Effect estimates were calculated using a random-effects model. RR and MDs were used for dichotomous and continuous outcomes, respectively, with 95% CIs. Heterogeneity was assessed using the Chi2 statistic (P < 0.01) and I2 statistic (> 50%).
2020_Wei	Role of neuromuscular blocking agents in acute respiratory distress syndrome: An updated meta-analysis of randomized controlled trials.	China	To assess the effect of NMBA on mortality, and better understand its use and adverse outcomes.	MEDLINE/Pubmed, Cochrane Library, WOS, ClinicalTrials.gov / July 2019	RoB 1	Effect of NMBA on ARDS outcomes assessed using RR and 95% CI. Random-effect model used if I-squared >50%. Publication bias and study heterogeneity evaluated using funnel plots, Egger's test, Q statistics, and I-squared.
2020_Yang3	Aminoglycoside use and intensive care unit-acquired weakness: A systematic review and meta-analysis.	China	To examine the relationship between aminoglycoside use and ICU-AW in critically ill patients.	MEDLINE/Pubmed, EMBASE, WOS, Cochrane CENTRAL, CINAHL / July 2019	Newcastle-Ottawa scale	They analyzed the results using ORs and 95% CIs, with DerSimonian and Laird random effects model for data analyses. We assessed the heterogeneity using the X^2 statistic with P < 0.1 considered statistically significant.
2018_Yang2	Risk factors for intensive care unit-acquired weakness: A systematic review and meta-analysis.	China	Summarize and incorporate the important risk factors from previously published multivariate analyses for ICUAW in critically ill adult patients.	MEDLINE, EMBASE, CENTRAL, China Biological Medicine, China National Knowledge Infrastructure, VIP and Wanfang databases / July 2017	Methodological assessment combined Downs and Black scale with two other prognostic models	M-A was conducted using the DerSimonian and Laird random-effects model to calculate the pooled effect size (OR, RR, HR) and its 95% CI. Heterogeneity was assessed using the I^2 statistic.

2018_Yang1	Corticosteroid use and intensive care unit-acquired weakness: a systematic review and meta-analysis.	China	To evaluated the relationship between corticosteroid use and ICU-acquired weakness in critically ill adult patients.	MEDLINE, EMBASE, Cochrane Central, WOS, CINAHL database / October 2017	Newcastle–Ottawa scale for prospective studies and RoB tool for RCTs	The results were analyzed using ORs and 95% CIs with the DerSimonian and Laird random effects model. Heterogeneity was assessed using the X^2 and I^2 statistics.
2018_Sanchéz-Solana	Acquired neuromuscular dysfunction in the intensive care unit.	España	Our secondary objective was to describe the association between this neuromuscular change and the ICU-LOS, the connection time to the MV, and the rate of failure in disconnection, consumption of corticosteroids and mortality.	MEDLINE, Scielo, WOS, Cochrane, CINAHL and Science Direct / Not informed	Levels of evidence (LE) and grades of recommendation (GR) following the SIGN scale	Not a meta-analysis / qualitative report of independent studies.
2018_Lambell	Association of energy and protein delivery on skeletal muscle mass changes in critically ill adults: A systematic review.	Australia	To examine the association of energy and/or protein provision on changes in skeletal muscle mas s in critically ill patie nts.	Ovid MEDLINE, EMBASE, CINAHL, Cochrane Central Register of Controlled Trials / March 2016	The ADA Quality Criteria Checklist for Primary Research (randomized and non-randomized nutrition studies)	Not a meta-analysis / qualitative report of independent studies.
2017_Annoni	Risk factors for intensive care acquired weakness: A systematic review and meta-analysis.	Brazil	We aimed to identify and synthetize the evidence about the prevalence, risk factors and outcomes of ICUAW in critically ill patients.	MEDLINE (Pubmed), CINAHL, EMBASE, PEDro, SciELO / 2007 to 2017	RoB	Not described
2017_McKittrick	Critical care polyneuropathy in burn injuries: An integrative review.	Australia	To identify factors that contribute to the development of critical care polyneuropathy in	PubMed, Scopus, CINHAL and EMBASE / July 2016	Not informed	Not a meta-analysis / qualitative report of independent studies.

			patients admitted to an ICU with a severe burn injury.			
2016_Price	Neuromuscular blocking agents and neuromuscular dysfunction acquired in critical Illness: A systematic review and meta-analysis.	USA	To examined the association between neuromuscular blocking agents and ICU-AW, CIP, and CIM.	MEDLINE (PubMed), EMBASE, WOS, CENTRAL, and CINAHL / September 2015	RoB for RCTs and the Newcastle-Ottawa Scale for prospective cohort studies	They calculate OR and perform M-A using the DerSimonian-Laird random effects model. Multivariate data were used when available. Heterogeneity was assessed using the I^2 statistic, with values below 40% indicating low heterogeneity.
2012_Ydemann	Treatment of critical illness polyneuropathy and/or myopathy - a systematic review.	Denmark	To search the literature with a view to providing a general description of critical illness myopathy/polyneuropathy (CIM/CIP), including its genesis and prevention.	PubMed, CINAHL and Swedmed+ / Not informed	Not informed	Not a meta-analysis / qualitative report of independent studies.
2010_Prentice	Differences in the degree of respiratory and peripheral muscle impairment are evident on clinical, electrophysiological and biopsy testing in critically ill adults: a qualitative systematic review.	Australia	To review original observational studies that measured an aspect of respiratory and peripheral muscle function in adults in the intensive care setting.	Scopus, PubMed, Ovid MEDLINE, CINAHL major citation databases / August 2009	Methodological Quality Instrument (MQI) for non-experimental designs	Not a meta-analysis, qualitative synthesis.
2006_Hohl	Critical illness polyneuropathy and myopathy: a review.	Switzerland	Are there tools available to assess the risk of a patient developing CIPNM? Is it possible to diagnose CIPNM early and to treat it before it appears clinically? What treatments are useful for CIPNM; especially nursing and physiotherapy interventions?	MEDLINE, Cochrane library, CINAHL / 1998 to 2006	Not informed	Not a meta-analysis / qualitative report of independent studies.

1998_De Jonghe	Acquired neuromuscular disorders in critically ill patients: a systematic review.	France	To summarize the prospective clinical studies of neuromuscular abnormalities in ICU patients.	MEDLINE, EMBASE, references in primary and review articles /1980 to September 1997	Others (validity of the study with data extraction)	Not a meta-analysis / qualitative report of independent studies.
*relevant for this overview and its outcomes SR: Systematic review, ICUAW: intensive care unit-acquired weakness, ICU: Intensive Care Unit, FiO2: fractional inspired oxygen, ARDS: acute respiratory distress syndrome, NMBA: neuromuscular blocking agents CIPNM: Patients critical illness polyneuromyopathy, CIP: critical illness polyneuropathy, CIM: critical illness myopathy, RCT: Randomized controlled trials, MV: mechanical ventilation, SIRS: Systemic Inflammatory Response Syndrome, MOF: multiple organ failure, MRC: Medical Research Council weakness scale, APACHE II: Acute Physiology and Chronic Health disease Classification System II, LOS: length of stay, 6MWD: 6-minute walk distance, CINAHL: Cumulative Index of Nursing and Allied Health Literature, WOS: Web of Science, CENTRAL: Cochrane Controlled Trials Registry, RCTs randomized controlled trials, RoB: Risk of Bias, CIs: Confiance Intervals, QUIPS: Quality in Prognosis Studies tool, QUADAS-2: Quality Assessment of Diagnostic Accuracy Studies criteria, OR: Odds ratios, RR: Relative risk, MDs: mean differences, ADA: American Dietetic Association, MQI: Methodological Quality Instrument						

Figure 1.
Overlap Citation Matrices for Each Factor Analyzed Across Multiple Meta-Analysis Reviews.

Figure 1.A

Ovelapping citation matrix of sex

**Study ID**	**Systematic Reviews**		
Primary Studies	2017_Annoni	2022_Yang_Zi	**N of times included**
2006_Khan	✖	■	1
2008_Nanas	✖	■	1
2010_Routsi	■	✖	1
2010_Sharshar	■	■	2
2011_Anastasopoulos	■	✖	1
2014_Fan	■	✖	1
2015_Conolly	■	✖	1
2015_Wieske	✖	■	1
2017_Dong		■	1
2017_Diaz		■	1
2018_Zhang		■	1
2019_Nie		■	1
2021_Raurell-Torredà		■	1
**Total studies included**	5	9	

■ Primary study included in the systematic reviews

Figure 1.B

Ovelapping citation matrix of severity of the pathology at ICU admission (APACHE II y III)

**Study ID**	**Systematic Reviews**		
Primary Studies	2018_Yang_2	2022_Yang_Zi	N of times included
2005_Amaya-Villar	✖	■	1
2005_Van Den Bergue	■	✖	1
2006_Khan	✖	■	1
2007_Hermans	■	✖	1
2008_Nanas	■	■	2
2011_Anastasopoulos	✖	■	1
2013_Hermans	■	✖	1
2014_Patel	■	✖	1
2016_Gupta	✖	■	1
2017_Diaz		■	1
2017_Dong		■	1
2018_Zhang		■	1
2019_Nie		■	1
**Total studies included**	5	9	

■ Primary study included in the systematic reviews

Figure 1.C

Ovelapping citation matrix of organ failure: SOFA

Study ID	Systematic Reviews			
Primary Studies	2017_Annoni	2018_Yang_2	2022_Yang_Zi	N of times included
2007_Hermans	✖	■	✖	1
2008_Ali	■	✖	✖	1
2008_Nanas	■	✖	■	2
2010_Weber-Carstens	■	■	✖	2
2011_Anastasopoulos	✖	✖	■	1
2014_Wieske	■	✖	✖	1
**Total studies included**	4	2	2	

■ Primary study included in the systematic reviews

SOFA: Sequential organ failure assessment

Figure. 1.D

Ovelapping citation matrix of sepsis

Study ID	Systematic Reviews			
Primary Studies	2017_Annoni	2018_Yang_1	2022_Yang_Zi	N of times included
2001_Garnacho-Montero	✗	■	✗	1
2005_Amaya-villar	✗	✗	■	1
2006_Khan	✗	■	✗	1
2010_Routsi	■	✗	✗	1
2014_Hermans	■	✗	✗	1
2016_Gupta	✗	■	✗	1
2016_Keh	✗	■	✗	1
2017_Diaz		✗	■	1
2019_Nie			■	1
**Total studies included**	2	4	3	

■ Primary study included in the systematic reviews

Supplemental material : Risk factors for intensive care unit-acquired weakness: an overview of systematic reviews and meta-analyses . Fuentes-Aspe, R.

Figure. 1.E

Ovelapping citation matrix use of mechanical ventilation

Study ID	Systematic Reviews			
Primary Studies	2018_Yang_1	2020_Medrinal	2022_Yang_Zi	N of times included
1998_Coakley	■	✖	✖	1
2001_deLetter	■	✖	✖	1
2001_Garnacho-Montero	■	✖	✖	1
2002_DeJongue	■	✖	✖	1
2005_Amaya-villar	✖	✖	■	1
2007_Lefaucheur	■	✖	✖	1
2007_Hermans	■	✖	✖	1
2008_Ali	■	■	✖	2
2010_Sharshar	■	✖	✖	1
2010_Brunello	■	■	✖	2
2010_Weber-Carstens	■	✖	✖	1
2011_Tzanis	✖	■	✖	1
2012_Lee	✖	■	✖	1
2014_Hermans	✖	■	✖	1
2014_Patel	■	✖	✖	1
2014_Wieske	■	✖	✖	1
2015_Wieske	✖	■	✖	1
2016_Gupta	✖	✖	■	1
2017_Diaz-Balive	✖	■	✖	1
2017_Medrinal	✖	■	✖	1
2017_Witeveen	✖	■	✖	1
2017_Dong	✖	✖	■	1
2017_Dres	✖	■	✖	1
2017_Carrie	✖	■	✖	1
2018_Zhang		✖	■	1
2019_Nie			■	1
**Total studies included**	12	11	5	

■ Primary study included in the systematic reviews

Supplemental material : Risk factors for intensive care unit-acquired weakness: an overview of systematic reviews and meta-analyses . Fuentes-Aspe, R.

Figure. 1.F

Ovelapping citation matrix use of corticosteroids

Study ID	Systematic Reviews			
**Primary Studies**	**2018_Yang_1**	**2018_Yang_2**	**2022_Yang_Zi**	**N of times included**
1998_Coakley	■	✕	✕	1
2001_deLetter	■	✕	✕	1
2001_Garnacho-Montero	■	✕	✕	1
2002_DeJongue	■	■	■	3
2005_Amaya-villar	✕	✕	■	1
2006_Khan	■	✕	■	2
2006_Lefaucheur	■	✕	✕	1
2007_Hermans	■	✕	✕	1
2008_Nanas	■	✕	■	2
2008_Ali	■	✕	✕	1
2010_Sharshar	■	✕	■	2
2010_Brunello	■	■	✕	2
2010_Weber-Carstens	■	✕	✕	1
2011_Anastosopoulos	■	✕	■	2
2013_Hermans	✕	■	✕	1
2014_Patel	■	✕	✕	1
2014_Wieske	■	✕	✕	1
2015_Nguyen	■	✕	✕	1
2016_Gupta	■	✕	■	2
2016_Keh	■	✕	✕	1
2017_Diaz	✕	✕	■	1
2019_Nie			■	1
**Total studies included**	18	3	9	

■ Primary study included in the systematic reviews

Figure. 1.G

Ovelapping citation matrix use of aminoglycosides

Study ID	Systematic Reviews			
**Primary Studies**	**2020_Yang_2**	**2020_Yang_3**	**2022_Yang_Zi**	**N of times included**
1997_Mohr	✗	■	✗	1
2001_deLetter	■	■	✗	2
2001_Garnacho-Montero	✗	■	✗	1
2002_DeJongue	✗	■	✗	1
2004_Khilnani	■	✗	✗	1
2005_Bercker	■	✗	✗	1
2005_VandenBergue	■	✗	✗	1
2005_Amaya-villar	✗	■	■	2
2007_Hermans	✗	■	✗	1
2008_Nanas	■	■	■	3
2010_Weber-Carstens	✗	■	✗	1
2011_Anastosopoulos	✗	■	■	2
2014_Wieske	■	■	✗	2
**Total studies included**	6	10	3	

■ Primary study included in the systematic reviews

Figure. 1.H

Primary Studies	2020_Yang_2	2022_Yang_Zi	N of times included
2001_Garnacho-Montero	■	✕	1
2007_Hermans	■	✕	1
2008_Nanas	✕	■	1
2013_Hermans	■	✕	1
2017_Dong	✕	■	1
2018_Zhang		■	1
2019_Nie		■	1
**Total studies included**	**3**	**4**	

■ Primary study included in the systematic reviews

RRT: Renal replacement therapy

Supplemental material : Risk factors for intensive care unit-acquired weakness: an overview of systematic reviews and meta-analyses . Fuentes-Aspe, R.

Figure 1. I

Ovelapping citation matrix for use of NMBA

Study ID	Systematics Reviews								
**Primary Studies**	**2016_Price**	**2017_Annoni**	**2018_Yang_2**	**2020_Shao**	**2020_Tarazan**	**2020_Wei**	**2022_Yang_Zi**	**2023_Bellaver**	**N of times included**
1992_Douglass	■	×	×	×	×	×	×	■	2
1992_Kupfer	×	×	×	×	×	×	×	■	1
1993_Coakley	■	×	×	×	×	×	×	■	2
1994_Verheul	■	×	×	×	×	×	×	×	1
1996_Leijten	■	×	×	×	×	×	×	■	2
1996_Leatherman	×	×	×	×	×	×	×	■	1
1999_Behbehani	×	×	×	×	×	×	×	■	1
2001_deLetter	■	×	×	×	×	×	×	■	2
2001_Garnacho-Montero	■	×	■	×	×	×	×	■	2
2002_DeJongue	■	×	×	×	×	×	■	■	3
2004_Gainner	×	×	×	×	■	■	×	■	3
2005_Garnacho-Montero	■	×	×	×	×	×	×	■	2
2005_Bednarik	■	×	×	×	×	×	×	×	1
2005_Amaya-villar	■	×	×	×	×	×	■	■	3
2006_Khan	×	×	×	×	×	×	■	×	1
2006_Forel	×	×	×	■	■	■	×	■	4
2007_DeJongue	■	×	×	×	×	×	×	×	1
2007_Hermans	■	×	■	×	×	×	×	■	3
2008_Nanas	■	×	■	×	×	×	×	■	3
2008_Ali	■	×	×	×	×	×	×	■	2
2009_Kesler	×	×	×	×	×	×	×	■	1
2010_Brunello	■	■	■	×	×	×	×	■	4
2010_Weber-Carstens	×	■	■	×	×	×	×	■	3
2010_Papazian	■	×	×	■	■	■	×	■	5
2011_Anastosopoulos	×	×	×	×	×	×	■	×	1
2012_Derde	■	×	×	×	×	×	×	×	1
2013_Hermans	×	×	■	×	×	×	×	×	1
2014_Hermans	■	■	×	×	×	×	×	■	3
2014_Fan	■	×	×	×	×	×	×	×	1
2014_Wieske	×	×	×	×	×	×	×	■	1
2015_Nguyen	×	■	■	×	×	×	×	■	3
2016_Gupta		×	×	×	×	×	■	■	2
2017_Diaz			×	×	×	×	■	×	1
2019_Moss				■	×	■	×	■	3
2019_Duan				×	■	×	×	×	1
2019_Nie				×	×	×	■	×	1
2021_Frithiof							×	■	1
2022_Núñez-Seisdedos								■	1
**Total studies included**	19	3	6	3	4	4	7	27	

■ Primary study included in the systematic reviews

NMBA use of neuromuscular blocking agents
